# Manipulating calcium homeostasis with nanoplatforms for enhanced cancer therapy

**DOI:** 10.1002/EXP.20230019

**Published:** 2023-10-10

**Authors:** Yanlin Feng, Jianlin Wang, Jimin Cao, Fangfang Cao, Xiaoyuan Chen

**Affiliations:** ^1^ Key Laboratory of Cellular Physiology at Shanxi Medical University, Ministry of Education, and the Department of Physiology Shanxi Medical University Taiyuan China; ^2^ Departments of Diagnostic Radiology, Surgery, Chemical and Biomolecular Engineering, and Biomedical Engineering, Yong Loo Lin School of Medicine and College of Design and Engineering National University of Singapore Singapore Singapore; ^3^ Clinical Imaging Research Centre, Centre for Translational Medicine, Yong Loo Lin School of Medicine National University of Singapore Singapore Singapore; ^4^ Nanomedicine Translational Research Program, NUS Center for Nanomedicine, Yong Loo Lin School of Medicine National University of Singapore Singapore Singapore; ^5^ Agency for Science, Technology, and Research (A*STAR) Institute of Molecular and Cell Biology Singapore Singapore

**Keywords:** Ca^2+^ inhibition, Ca^2+^ overload, calcium homeostasis regulation, cancer therapy, immunotherapy

## Abstract

Calcium ions (Ca^2+^) are indispensable and versatile metal ions that play a pivotal role in regulating cell metabolism, encompassing cell survival, proliferation, migration, and gene expression. Aberrant Ca^2+^ levels are frequently linked to cell dysfunction and a variety of pathological conditions. Therefore, it is essential to maintain Ca^2+^ homeostasis to coordinate body function. Disrupting the balance of Ca^2+^ levels has emerged as a potential therapeutic strategy for various diseases, and there has been extensive research on integrating this approach into nanoplatforms. In this review, the current nanoplatforms that regulate Ca^2+^ homeostasis for cancer therapy are first discussed, including both direct and indirect approaches to manage Ca^2+^ overload or inhibit Ca^2+^ signalling. Then, the applications of these nanoplatforms in targeting different cells to regulate their Ca^2+^ homeostasis for achieving therapeutic effects in cancer treatment are systematically introduced, including tumour cells and immune cells. Finally, perspectives on the further development of nanoplatforms for regulating Ca^2+^ homeostasis, identifying scientific limitations and future directions for exploitation are offered.

## Graphical abstract

This review paper provides a comprehensive overview of nanoplatforms that manipulate calcium ions (Ca^2+^) homeostasis to enhance cancer therapy. It offers a detailed exploration of two Ca^2+^ regulation strategies employed by these nanoplatforms, including Ca^2+^ overload and inhibition, and discusses the applications of Ca^2+^‐related nanomaterials in various cell types, such as cancer cells and immune cells. The paper also presents perspectives on further advancing nanoplatforms for regulating Ca^2+^ homeostasis, identifying scientific limitations, and outlining future directions for exploration.

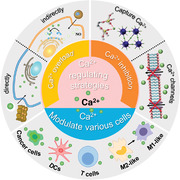

## 1 INTRODUCTION AND BACKGROUND

As the most abundant second messenger in the human body, calcium (Ca^2+^) plays a diverse and substantial role in numerous biological processes, such as gene expression, cell cycle regulation, cell proliferation, autophagy and apoptosis.^[^ ^1^ ^]^ To achieve specific cellular outcomes, Ca^2+^ homeostasis is maintained by the coordinated regulation of Ca^2+^ channels, Ca^2+^ pumps, transporters, and exchangers.^[^ ^2^, ^3^, ^4^, ^5^ ^]^ Abnormal levels of Ca^2+^ have been closely linked to a variety of diseases including tumours, cardiovascular disease, neurodegenerative disorders, hypertension, and diabetes.^[^ ^6^, ^7^, ^8^ ^]^ Notably, cancer cells exhibit greater sensitivity to Ca^2+^ regulation than normal cells during tumourigenesis and tumour progression. Disruption of Ca^2+^ homeostasis can lead to irreversible cell damage and even cell death, making it a topic of increasing interest in recent years. Firstly, Ca^2+^ is the most abundant metal ion in the human body and plays a crucial role in regulating various cellular processes such as proliferation, differentiation, metabolism, and cell death. Secondly, the preparation method of Ca^2+^‐based nanomaterials is simple, cost‐effective, and biosecure, with degradation products that are harmless to human beings. Thirdly, by utilizing endogenous Ca^2+^, regulatory Ca^2+^‐mediated cancer therapies can selectively trigger tumour cell death without relying on exogenous cytotoxic drugs. Moreover, Ca^2+^ homeostasis‐based therapies do not require external stimuli like photothermal therapy (PTT), photodynamic therapy (PDT), and sonodynamic therapy (SDT), favouring a wide range of biomedical applications and clinical translation. Therefore, regulating Ca^2+^ homeostasis is crucial for the treatment of malignant tumours among various therapeutic approaches.

With the rapid advancement of nanoscience and nanotechnology in biomedicine, Ca‐containing nanomaterials have demonstrated immense potential for disease therapy.^[^ ^9^, ^10^, ^11^, ^12^, ^13^, ^14^ ^]^ These materials have garnered significant attention due to their ability to directly release Ca^2+^ into cells and regulate intracellular Ca^2+^ content.^[^ ^15^ ^]^ The majority of the Ca^2+^ present in these nanoparticles exists as biominerals, such as calcium carbonates (CaCO_3_), calcium peroxide (CaO_2_), calcium hydride (CaH_2_), calcium phosphates (CaP), calcium fluoride (CaF_2_) and so on. They demonstrate exceptional biocompatibility, bioactivity and biodegradability, making them promising platforms for delivering small molecules that regulate Ca^2+^ homeostasis or directly transport Ca^2+^ to modulate its content.

**FIGURE 1:**
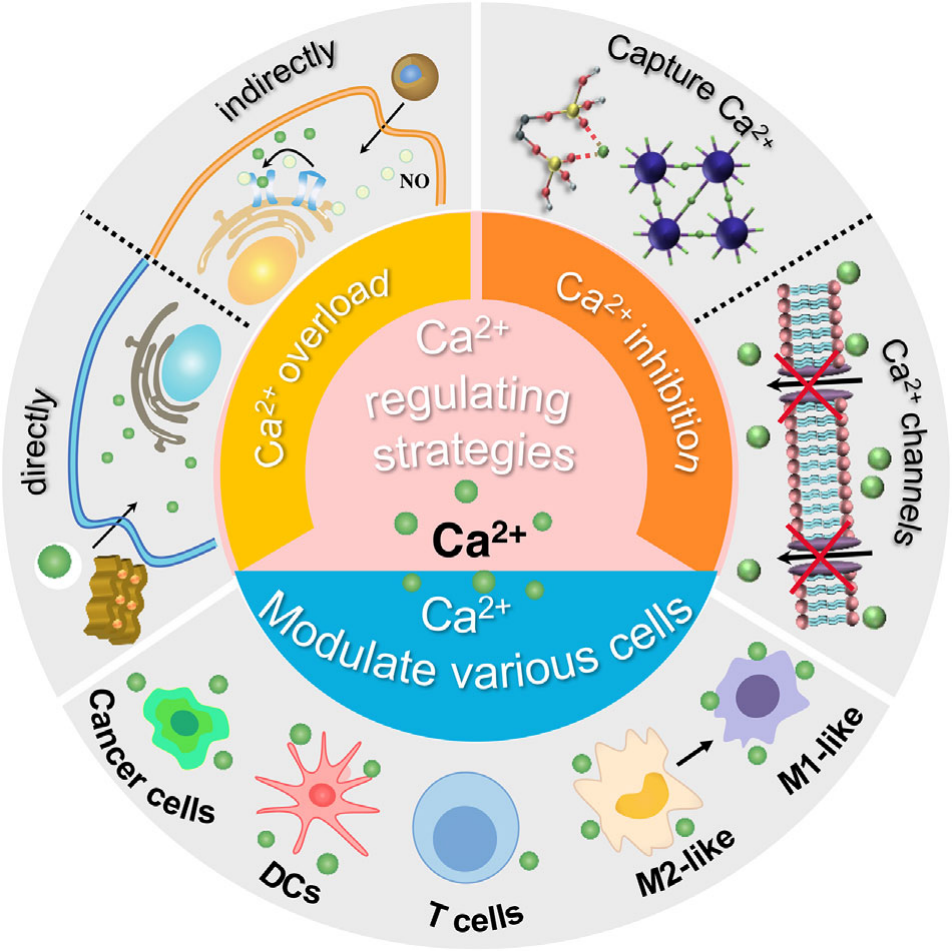
Schematic illustration of different strategies for regulating Ca^2+^ balance in cancer treatment (Ca^2+^ overload/inhibition), and the applications of Ca^2+^‐related nanomaterials in different cells (cancer cells, immune cells) to regulate their Ca^2+^ balance for achieving therapeutic effects in cancer treatment.

Ca^2+^‐based nanomaterials have been utilized as disruptors of Ca^2+^ homeostasis, either by inducing Ca^2+^ overload to amplify cancer therapy or by reducing Ca^2+^ levels to reverse drug resistance in tumours.^[^ ^16^, ^18^ ^]^ Specifically, Ca^2+^ overload can lead to mitochondrial dysfunction, elevated reactive oxygen species (ROS) level, cellular and organelle membrane damage and cytoskeletal destruction, thus causing cancer cell death.^[^ ^19^, ^20^ ^]^ Conversely, high levels of Ca^2+^ in tumours can induce drug resistance,^[^ ^21^ ^]^ thereby reducing Ca^2+^ levels in a controlled manner using Ca^2+^ antagonists and small interfering RNA (siRNA) can reverse this drug resistance.^[^ ^22^, ^23^, ^24^ ^]^ Decreasing Ca^2+^ concentration can decrease mitochondrial membrane potential (ΔΨ), promote apoptosis of tumour cells, inhibit tumour metastasis, and sensitize drug‐resistant tumours to chemotherapy drugs.^[^ ^25^, ^26^ ^]^ Furthermore, Ca^2+^‐based nanomaterials can also be used to intervene in the Ca^2+^ homeostasis of immune cells, augmenting immunity and promoting cancer immunotherapy.^[^ ^27^, ^28^, ^29^, ^30^ ^]^ In this review, we systematically introduce nanoplatforms as Ca^2+^ homeostasis disruptors for cancer therapy. We provide an overview of diverse strategies and materials employed in the regulation of Ca^2+^ balance for cancer treatment. These include substances that directly or indirectly induce Ca^2+^ overload or Ca^2+^ inhibition. Subsequently, we delve into the application of these materials for targeting various cell types, aiming to modulate their Ca^2+^ balance and achieve therapeutic effects in cancer treatment. This encompasses both tumour cells and immune cells (Figure 1). Additionally, we address the challenges and potential avenues for future research in Ca^2+^‐based nanotherapies. We intend to stimulate the development of novel nanoplatforms and present an optimistic outlook for disease treatment.

## 2 NANOPLATFORM AS A POTENTIAL CA^2+^ HOMEOSTASIS DESTROYER

### 2.1 Ca^2+^ overload

Ca^2+^ overload is characterized by the aberrant accumulation of Ca^2+^ in the cytoplasm, which can cause various types of cellular damage and even death. In recent years, Ca^2+^‐based biomineralized nanomaterials, such as CaCO_3_, CaO_2_, CaH_2_, CaP, and CaF_2_ have been widely developed for biomedical applications due to their biodegradability and biocompatibility. Ca^2+^ overload could be directly achieved by using the Ca^2+^‐based biomineralized nanomaterials or other nanomaterials capable of loading Ca^2+^. However, excessive use of exogenous Ca^2+^ can potentially lead to an acute rise in plasma Ca^2+^ levels, resulting in a sharp decrease in blood pH and an increase in the inflammatory reaction.^[^ ^31^, ^32^ ^]^ Therefore, non‐Ca^2+^ nanomaterials could also be used to indirectly regulate Ca^2+^ overload. For instance, plasma membrane damage can trigger extracellular Ca^2+^ influx, causing intracellular Ca^2+^ overload and inducing cell death. Another approach involves the utilization of nitric oxide (NO) to activate the overexpressed ryanodine receptor (RyR) channels in cancer cells, leading to abrupt Ca^2+^ elevation and intracellular Ca^2+^ overload, which ultimately induces cell apoptosis.

#### 2.1.1 Nanoplatform‐derived Ca^2+^ directly triggers Ca^2+^ overload

Most of the nanomaterials utilized in Ca^2+^ overload‐based cancer therapy are Ca^2+^‐based nanomaterials. They can specifically accumulate at the tumour site via passive targeting and active targeting. Then, they can react with hydrogen ion (H^+^) to release Ca^2+^ in the tumour environment, participating in and inducing Ca^2+^ overload to kill the cancer cells. These Ca^2+^‐based nanomaterials have demonstrated numerous advantages, including excellent biocompatibility, long‐term biodegradability, biosafety, and efficacy, rendering them highly promising candidates for tumour treatment.

##### CaCO_3_

**FIGURE 2:**
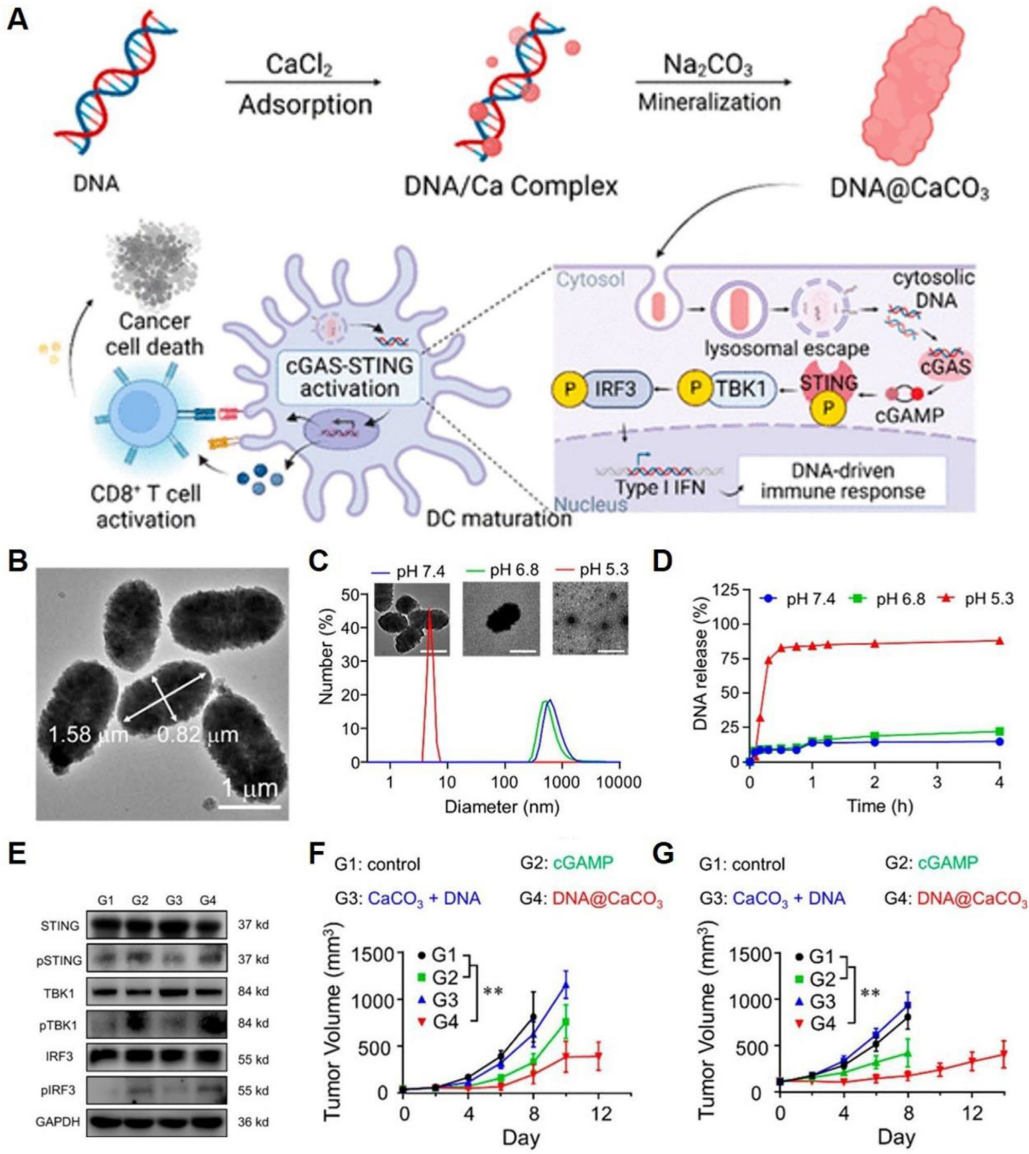
CaCo_3_ as intelligent carrier to deliver different types of cargos. (A) Scheme illustration of the biomineralization processes of DNA@CaCO_3_ with cGAS‐STING activating abilities to inhibit tumour recurrence. (B) TEM image of DNA@CaCO_3_ microparticles. (C) pH‐responsive decomposition properties of DNA@CaCO_3_ microparticles and (D) time‐dependent release curves of DNA from DNA@CaCO_3_ microparticles. (E) Western blotting of the proteins correlated with cGAS‐STING‐TBK1‐IRF3 signalling pathways. (F) Tumour growth curves of B16 tumours in C57BL/6 mice and (G) CT26 tumours in BALB/c mice. Reproduced with permission.^[^ ^39^ ^]^ Copyright 2023, American Chemical Society.

It is well acknowledged that CaCO_3_, a naturally occurring material, exhibits excellent biocompatibility and biodegradability. Its stability at neutral pH and susceptibility to decomposition in the acidic tumour microenvironment (TME) render it an ideal intelligent carrier to deliver different types of cargos.^[^ ^33^, ^34^, ^35^, ^36^, ^37^, ^38^ ^]^ Recently, Li et al. developed pH‐responsive dsDNA‐loaded CaCO_3_ (DNA@CaCO_3_) microparticles by biomineralization, utilizing the strong affinity between Ca^2+^ and dsDNA (Figure 2A).^[^ ^39^ ^]^ These DNA@CaCO_3_ microparticles exhibited enhanced stability, retaining their nanogranular structure and dispersity compared to conventional CaCO_3_ microparticles obtained through traditional mixing of calcium chloride (CaCl_2_) and sodium carbonate (Na_2_CO_3_) solutions (Figure 2B). The DNA@CaCO_3_ microparticles displayed pH‐dependent decomposition properties to release DNA (Figure 2C,D) and activate the cGAS‐STING‐TBK1‐IRF3 pathway (Figure 2E). Consequently, they effectively reversed tumour immunosuppression, inhibiting tumour growth in both murine B16 and CT26 tumours in immune‐competent C57BL/6 and BALB/c mice (Figure 2F,G).

**FIGURE 3:**
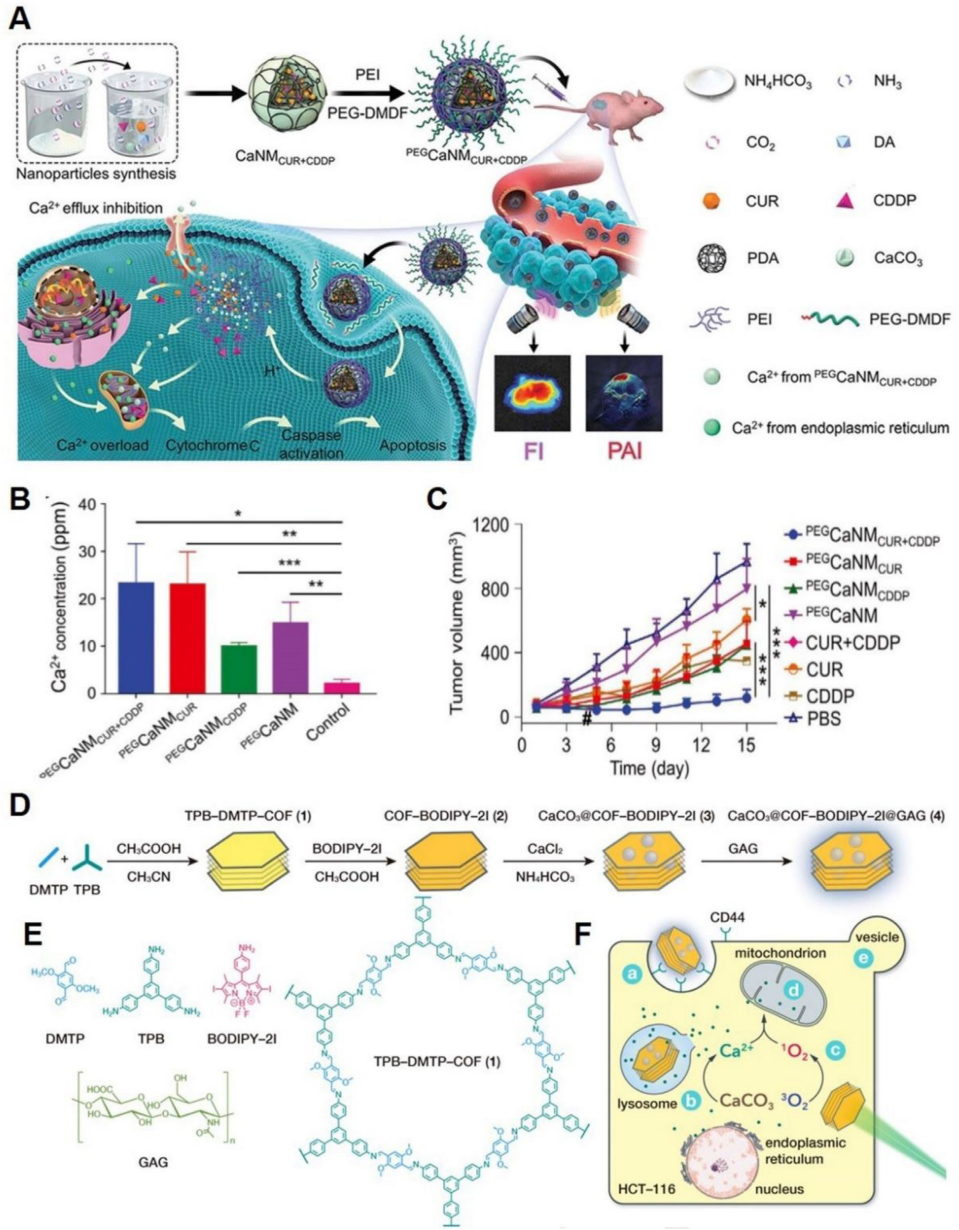
CaCO_3_ based nanoplatform for Ca^2+^ overload. (A) Scheme of CaCO_3_@PDA@CUR@CDDP‐based synergistic Ca^2+^ overload and chemotherapy. (B) Mitochondrial Ca^2+^ concentrations quantification after treated with various Ca^2+^ nanomodulators. (C) Tumour growth curves of MCF‐7‐tumour‐bearing nude mice. Reproduced with permission.^[^ ^45^ ^]^ Copyright 2021, John Wiley & Sons. (D) Schematic diagram of the synthesis process of CaCO_3_@COF‐BODIPY‐2I@GAG NMs. (E) Molecular structure of DMTP, TPB, BODIPY‐2I, GAG, AND TPB‐DMTP‐COF (1). (F) Synergistic induction of intracellular Ca^2+^ overload by singlet oxygen (^1^O_2_) and exogenous Ca^2+^ delivery. Note: (A) CD44‐mediated cellular uptake; (B) CaCO_3_ decomposition in lysosomes; (C) BODIPY‐2I induce ^1^O_2_ production under green LED. (D) Mitochondrial impairment. (E) Cell blebbing induced by oncosis. Reproduced with permission.^[^ ^46^ ^]^ Copyright 2020, John Wiley & Sons.

On the other hand, CaCO_3_ poses minimal risk of adverse effects as its degraded byproducts comprise only Ca^2+^ and carbon dioxide (CO_2_). The former can be eliminated from the body via renal excretion or deposited in bones while the latter can be exhaled by the lungs.^[^ ^40^, ^41^ ^]^ CaCO_3_ has shown promise as a Ca^2+^ donor for achieving Ca^2+^ overload in biological therapy. However, using CaCO_3_ NPs alone is not sufficient to achieve satisfactory therapeutic outcomes due to cells’ ability to self‐regulate intracellular Ca^2+^ concentration. Cells possess mechanisms such as the plasma membrane calcium pump, mitochondria, and endoplasmic reticulum (ER) to regulate and buffer intracellular Ca^2+^ levels.^[^ ^42^, ^43^ ^]^ These mechanisms can pump out excess Ca^2+^ or absorb Ca^2+^ to maintain cellular homeostasis. To disrupt cellular calcium buffering and promote Ca^2+^ overload, it is necessary to impair the capacity of mitochondria or ER to regulate Ca^2+^ levels. This can be achieved by damaging these organelles. Additionally, the efflux of Ca^2+^ can be inhibited by using curcumin (CUR), a Ca^2+^ channel inhibitor.^[^ ^44^ ^]^ Zheng et al. developed a nanoplatform for Ca^2+^‐overload‐dominated cancer therapy using CaCO_3_ nanocarriers co‐loaded with CUR and cisplatin (CDDP), which were prepared using polydopamine (PDA) as a template (Figure 3A).^[^ ^45^ ^]^ This nanoplatform caused multilevel mitochondrial impairment by inducing Ca^2+^ overload. Specifically, a massive amount of Ca^2+^ was released from CaCO_3_ component to achieve intramitochondrial Ca^2+^ overload (Figure 3B); Subsequently, the released CUR further promoted Ca^2+^ release from the ER to the cytoplasm and inhibited Ca^2+^ efflux. Lastly, CDDP destructed the mitochondrial structure and metabolic function, resulting in mitochondrial dysfunction and final tumour inhibition (Figure 3C). Additionally, the nanoplatform was endowed with dual imaging capabilities of photoacoustic (PA) and fluorescence using PDA and CUR, respectively. The integration of imaging and treatment holds immense potential for advancing cancer therapy. In another work, Dong et al. developed CaCO_3_@COF‐BODIPY‐2I@GAG, which consists of a covalent organic framework (COF), a photosensitizer‐modified BODIPY2I, glycosaminoglycans (GAGs), and nano‐CaCO_3_ (Figure 3D,E).^[^ ^46^ ^]^ The use of nano‐CaCO_3_ ensured the safe delivery of drugs to the TME without premature leakage. Upon reaching lysosomes with pH = 5.0, the nano‐CaCO_3_ broke down and released Ca^2+^ to induce Ca^2+^ overload. Furthermore, when exposed to light, CaCO_3_@COF‐BODIPY‐2I@GAG demonstrated PDT performance, destroying cell Ca buffering capacity and amplifying the cell damage in vitro and in vivo caused by Ca^2+^ overload (Figure 3F).

##### CaO_2_

Currently, CaO_2_ is widely used in disinfection and degradation of contaminants. As a solid, it is sometimes referred to as “solid hydrogen peroxide (H_2_O_2_)” as it produces H_2_O_2_ when reacting with H_2_O. The reaction between CaO_2_ and H_2_O depends on the pH of the reaction system, enabling them to instantaneously decompose into Ca^2+^ and H_2_O_2_:^[^ ^47^ ^]^

$${\mathrm{CaO}}_{2}+2H_{2}O\overset{H^{+}}{\to }\mathrm{Ca}{\left(\mathrm{OH}\right)}_{2}+H_{2}O_{2}$$

$${\mathrm{CaO}}_{2}+H_{2}O\to \mathrm{Ca}{\left(\mathrm{OH}\right)}_{2}+\frac{1}{2}O_{2}$$

**FIGURE 4:**
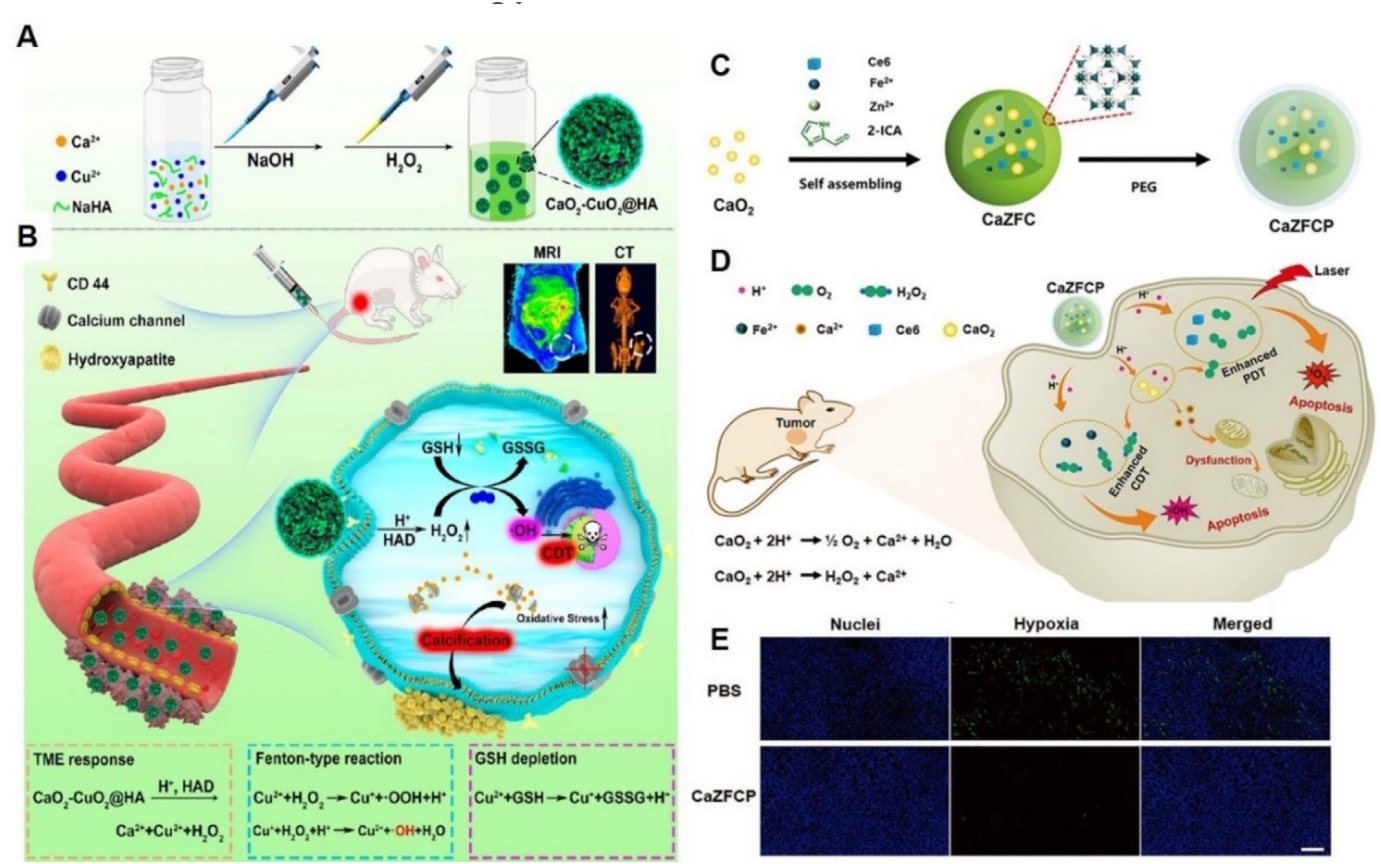
CaO_2_‐based nanoplatform for Ca^2+^ overload and CDT. (A) Schematic illustration of the synthesis process of CaO_2_‐CuO_2_@HA NC and (B) antitumour properties of CaO_2_‐CuO_2_@HA NC for Ca^2+^ overload and CDT. Reproduced with permission.^[^ ^54^ ^]^ Copyright 2022, American Chemical Society. (C) Schematic illustration of the synthesis process of CaZFCP and (D) therapeutic mechanism of pH‐responsive CaZFCP for Ca^2+^ overload, CDT and PDT. (E) Hypoxia‐inducible factor‐1α (HIF‐1α) staining of tumour tissues after treated with PBS, ZFCP, and CaZFCP. Reproduced with permission.^[^ ^55^ ^]^ Copyright 2021, John Wiley & Sons.

CaO_2_ could induce direct oxidative stress through Ca^2+^ overload and H_2_O_2_ production, ultimately leading to cell death.^[^ ^48^, ^49^, ^50^ ^]^ Additionally, the generated H_2_O_2_ can inhibit Ca^2+^ efflux, enhancing the Ca^2+^ overload efficiency. Moreover, because CaO_2_ could produce a high level of H_2_O_2_, it is a promising strategy to combine CaO_2‐_based Ca^2+^ overload with chemodynamic therapy (CDT), which could catalyze H_2_O_2_ into toxic hydroxyl radical (•OH) through Fenton or Fenton‐like reactions.^[^ ^51^, ^52^, ^53^ ^]^ Liu and co‐workers prepared a sodium hyaluronate (HA) modified CaO_2_ and copper peroxide (CuO_2_) nanocomposite (denoted as CaO_2_–CuO_2_@HA NC) for tumour therapy (Figure 4A,B).^[^ ^54^ ^]^ CaO_2_–CuO_2_@HA NC could mitigate the release of H_2_O_2_ under physiological conditions and undergo rapid decomposition upon reaching the tumour site due to its acidic microenvironment. Cu^2+^ could act as a catalyst to facilitate the generation of abundant •OH from self‐supplying H_2_O_2_ through the Fenton‐type reaction, while simultaneously depleting the overexpressed GSH through a redox reaction, thus further enhancing CDT. Overloaded Ca^2+^ caused mitochondria injury, which further exacerbated the oxidative stress in tumour cells. In addition, the therapeutic process could be effectively monitored through dual supervision of Cu^2+^‐induced magnetic resonance imaging (MRI) and Ca^2+^‐overloading‐promoted computed tomography (CT) imaging. In short, CaO_2_ represents a breakthrough in the development of Ca^2+^‐based nanomaterials for tumour treatment, as it not only synergistically harnesses the dual functions of Ca^2+^ and H_2_O_2_ to induce apoptosis but also serves as a mediator of Ca^2+^ overload and Cu‐mediated CDT, both of which rely on the important role of intracellular ions in cell function. Furthermore, due to the high level of oxygen (O_2_) produced by CaO_2_, CaO_2_‐based composites could serve as a source of O_2_ to improve PDT efficiency. Shen et al. developed a CaO_2_@ZIF‐Fe/Ce6@PEG (CaZFCP) composite to simultaneously achieve the Ca^2+^ overload, CDT and PDT (Figure 4C,D).^[^ ^55^ ^]^ The generated O_2_ from CaO_2_ could alleviate the hypoxic tumour environment, thereby improving the efficacy of PDT by producing more ^1^O_2_ (Figure 4E). This approach holds promise for enhancing the therapeutic outcomes of PDT by addressing the hypoxia‐related limitations in tumour treatment.

Besides, CaO_2_ could also be utilized as a drug carrier for loading drugs that destroy cellular Ca^2+^ buffering capacity, promote Ca^2+^ influx, or inhibit Ca^2+^ efflux, thereby enhancing the Ca^2+^ overload efficiency.^[^ ^56^, ^57^ ^]^ For instance, it was found that the Transient Receptor Vanilloid 1 (TRPV1) thermosensitive cation channel could promote Ca^2+^ influx when the temperature exceeds 43°C. Based on this finding, Zhou and colleagues designed a near‐infrared (NIR)‐responsive DPPC‐DSPE‐PEG2000‐NH_2_@PDPP@CaO_2_@doxorubicin NPs to acquire chemotherapy, Ca^2+^ overload and PTT.^[^ ^58^ ^]^ With NIR irradiation, the PTT effect could elevate the local temperature at the tumour site, activating the TRPV1 channel and promoting Ca^2+^ influx to achieve more efficient Ca^2+^ overload therapy.

##### CaH_2_

CaH_2_ is a portable hydrogen (H_2_) source that has shown promising results in biomedical applications in recent years. Upon reaction with H_2_O, CaH_2_ produces Ca^2+^, H_2_, and hydroxide ions (OH^−^). The resulting Ca^2+^ can induce Ca^2+^ overload, which can trigger various cellular processes, while OH^−^ can neutralize the acidic TME. Additionally, the high efficiency of H_2_ can also trigger H_2_ therapy, which can inhibit the energy supply of cancer cells, downregulate the expression of vascular endothelial growth factor in tumours, and elicit a systemic immune response.^[^ ^59^ ^]^ Therefore, CaH_2_ has great potential as a multifunctional nanoplatform for cancer treatment.

**FIGURE 5:**
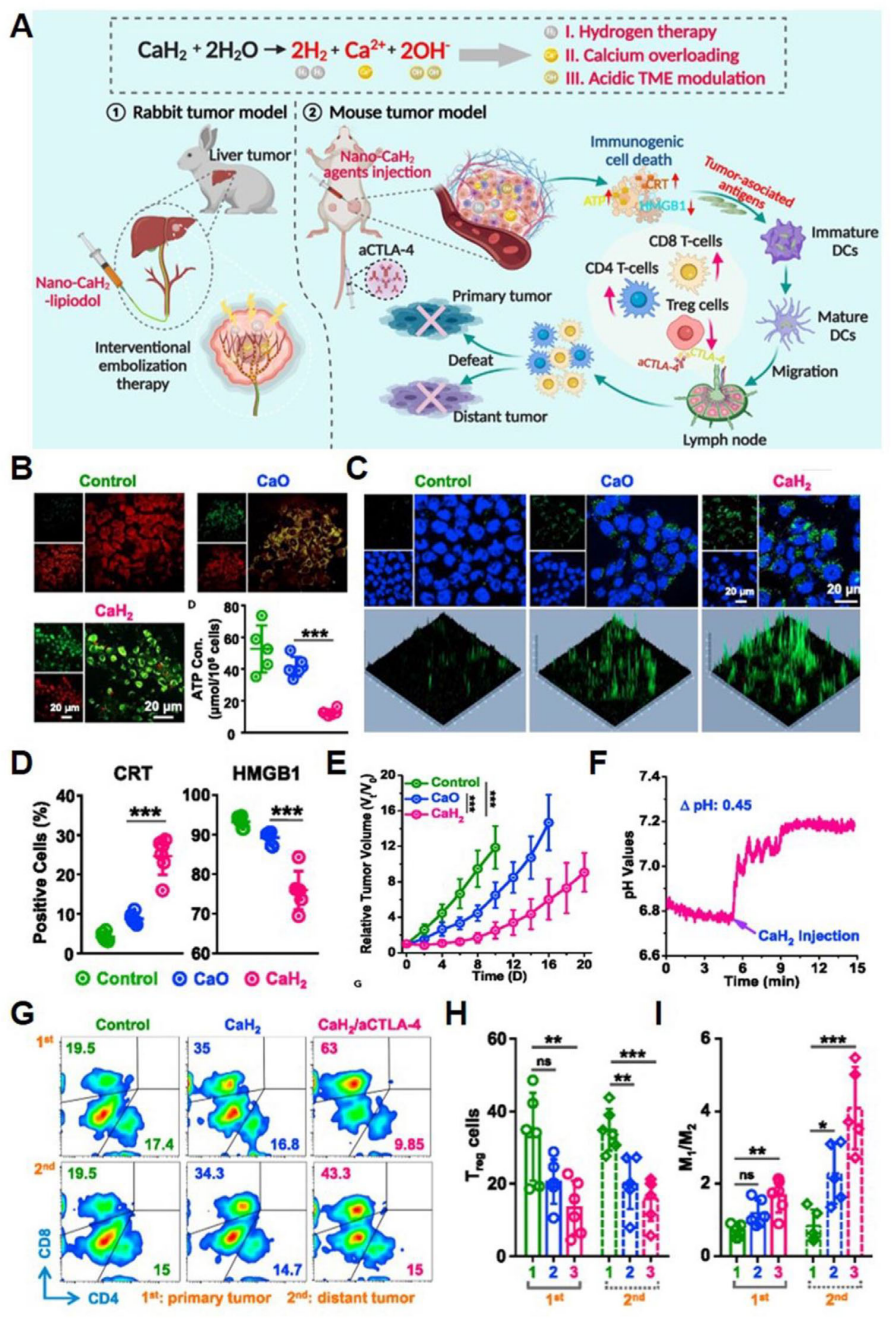
CaH_2_‐based nanoplatform for Ca^2+^ overload, H_2_ therapy and acidic TME modulation. (A) Schematic illustration of the application of nano‐CaH_2_ for mouse xenograft and interventional transarterial embolization (TAE) therapy of rabbit liver tumours through the combination of Ca^2+^ overload, H_2_ immunotherapy and neutralization of acidic tumour microenvironment. (B) Mitochondrial dysfunction and ATP generation inhibition. (C) Intracellular Ca^2+^ content after different treatment. (D) characterization of ICD. (E) Tumour volume monitoring. (F) pH values inside the tumours. (G) CTL infiltration, (H) quantification of Treg cells, and (I) the polarization of macrophages within the primary and distant tumours after different treatments. Reproduced with permission.^[^ ^60^ ^]^ Copyright 2022, Elsevier.

Due to the functions of H_2_ and Ca^2+^ in antitumour therapies, respectively, Gong and co‐workers designed and synthesized CaH_2_ nanomaterials using liquid‐phase exfoliation technology and employed them as a type of anticancer agent (Figure 5A).^[^ ^60^ ^]^ The nano‐CaH_2_ was modified with PEG200 to enhance dispersion, and then employed to react with H_2_O to produce H_2_ and Ca^2+^ and OH^−^. In vitro experiments showed that CaH_2_ induced mitochondrial dysfunction and decreased adenosine 5′‐triphosphate (ATP) content (Figure 5B), which ascribe to the increased intracellular Ca^2+^ level (Figure 5C). Moreover, CaH_2_ could trigger strong immunogenic cell death (ICD) effects for colon and breast cancer cells (Figure 5D). Therefore, local injection of nano‐CaH_2_ could effectively eradicate tumours (Figure 5E) through combination therapy including Ca^2+^ overload, H_2_ therapy, TME regulation and immune system activation. Increased pH value inside the tumour was observed after CaH_2_ treatment (Figure 5F). Combined with cytotoxic T‐lymphocyte‐associated antigen 4 (CTLA‐4) checkpoint, nano‐CaH_2_ effectively eradicates both primary and distant tumours. The CaH_2_/CTLA‐4 group could increase the cytotoxic T lymphocyte (CTL) infiltration (Figure 5G), decrease the percentage of Treg cells (Figure 5H) and promote the polarization of macrophages to M1 type (Figure 5I) in both tumours, leading to a robust immune response. In vivo interventional embolization studies demonstrated that nano‐CaH_2_ could alleviate tumour hypoxia and metastasis. In addition, when dispersed in lipiodol to obtain CaH_2_‐lipiodol dispersion, they demonstrated promising potential for enhancing the therapeutic effect of transarterial embolization treatment of liver cancer in rabbits compared to that used in situ lipiodol alone.^[^ ^61^ ^]^ As a metal hydride material, nano‐CaH_2_ plays a significant role in cancer therapy by enabling H_2_ therapy and inducing anti‐tumour immunity. Moreover, its degradation products of Ca^2+^, OH^−^, and H_2_, do not have any long‐term adverse effects on the body, therefore, nano‐CaH_2_ holds significant potential for future clinical applications.

##### CaP

**FIGURE 6:**
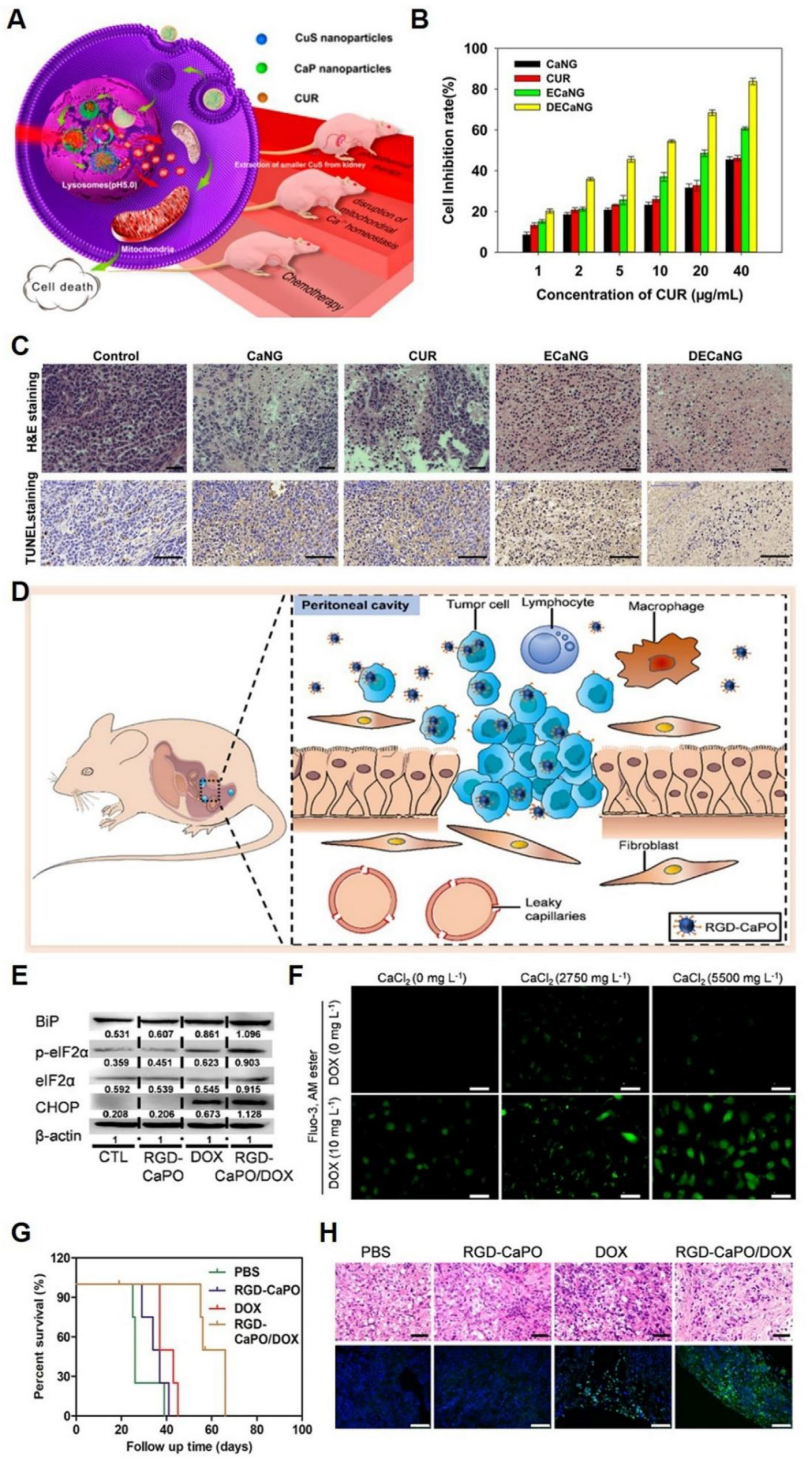
CaP‐based nanoplatform for Ca^2+^ overload. (A) Schematic illustration of dual enhanced Ca^2+^ nanogenerator (DECaNG) synergized with Ca^2+^ overload and PTT. DECaNG induces tumour cell apoptosis both (B) in vitro and (C) in vivo. Reproduced with permission.^[^ ^70^ ^]^ Copyright 2018, American Chemical Society. (D) Schematic illustration of the anti‐tumour mechanism of RGD‐CaPO/DOX in tumour cells and peritoneal cavity of mice. (E) Western blotting of the proteins correlated with endoplasmic reticulum stress. (F) Fluorescent images to detection the intracellular Ca^2+^. (G) Survival proportions of mice after different treatments. (H) Hematoxylin−eosin (H&E) and terminal deoxynucleotidyl transferase dUTP nick end labelling (TUNEL) images of tumour tissues after different treatments. Reproduced with permission.^[^ ^67^ ^]^ Copyright 2022, American Chemical Society.

CaP is a promising nanocarrier for Ca^2+^ release, due to its good biosafety, bioactivity, and biodegradability.^[^ ^62^ ^]^ It has been extensively employed in the fields of bone tissue engineering and tumour treatment.^[^ ^63^, ^64^, ^65^, ^66^, ^67^, ^68^ ^]^ What's more, CaP nanoparticles have been found to release abundant Ca^2+^ in an acidic pH environment,^[^ ^69^ ^]^ making them one of the most promising Ca^2+^‐based materials. Xu et al. developed a dual‐enhanced Ca^2+^ nanogenerator (DECaNG) that triggers intracellular Ca^2+^ elevation through three distinct pathways (Figure 6A).^[^ ^70^ ^]^ Firstly, they used CaP‐doped hollow mesoporous copper sulphide (CuS) as the basic Ca^2+^ nanogenerator that directly and persistently releases Ca^2+^ responding to the lower pH in TME. Secondly, NIR light radiation could disturb the crystal lattice of hollow mesoporous CuS, leading to accelerated Ca^2+^ generation from the nanocarriers. Finally, CUR could promote Ca^2+^ release from ER to the cytoplasm and inhibit the outflow of Ca^2+^ in the cytoplasm, thereby causing excessive Ca^2+^ flow into mitochondria, disrupting mitochondrial Ca^2+^ homeostasis and resulting in cell apoptosis (Figure 6B). The nanoplatform exhibited excellent tumour targeting and cancer cell apoptosis in vivo (Figure 6C) by disrupting mitochondrial Ca^2+^ homeostasis and PTT. Furthermore, the metabolism of CaNG could be accelerated through its breakdown into smaller nanoparticles, thereby shortening the retention time of the nanoplatform in vivo. The CaP‐mediated Ca^2+^ overload can be used in conjunction with other cancer treatments, making it a promising strategy for tumour treatment. In addition to their ability to regulate Ca^2+^ levels, amorphous CaP nanosystems can also be utilized for drug delivery in the treatment of cancers. Qiu et al. designed an amorphous CaP nanosystem modified with Arg‐Gly‐Asp (RGD) and loaded doxorubicin (RGDCaPO/DOX) for disseminated ovarian cancer therapy (Figure 6D).^[^ ^67^ ^]^ The RGDCaPO/DOX nanosystem exhibited its antitumour effect through two primary mechanisms: the exacerbation of ER stress (Figure 6E) and the induction of Ca^2+^ overload (Figure 6F). The nanosystem was administered via intraperitoneal injection, and this delivery method resulted in a positive antitumour effect (Figure 6G,H).

##### CaS

**FIGURE 7:**
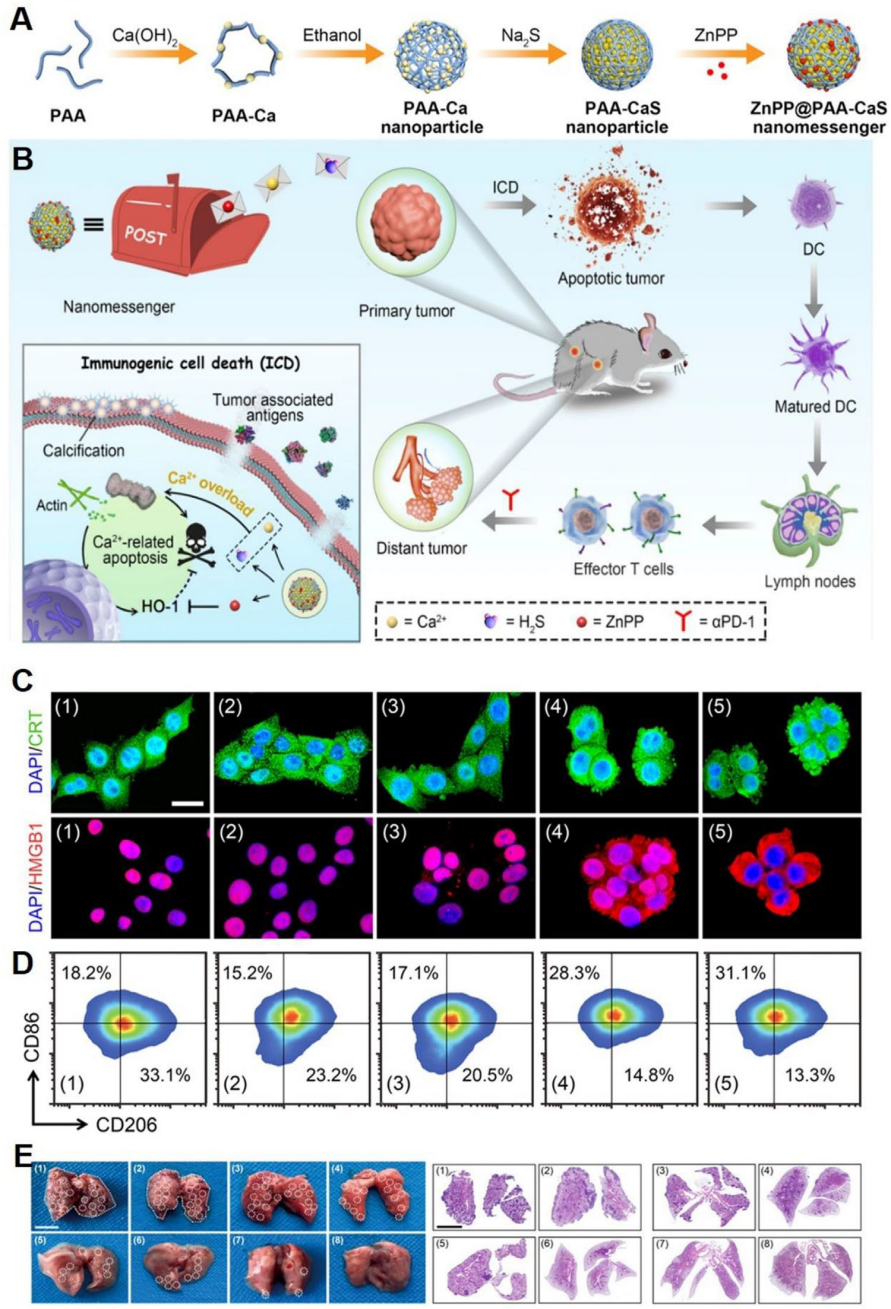
CaS‐based nanoplatform for Ca^2+^ overload. (A) Schematic illustration of the preparation process of ZnPP@PAA‐CaS and (B) ZnPP@PAA‐CaS mediated Ca^2+^ overload and signalling cascade antitumour immunotherapy. (C) characterization of ICD. (D) The polarization of macrophages. (1) blank; (2) PAA‐Ca; (3) ZnPP; (4) PAA‐CaS; (5) ZnPP@PAA‐CaS. (E) Digital photos of metastatic nodules in lungs after different treatments and corresponding H&E staining images. All groups: (1) blank; (2) PAA‐Ca; (3) ZnPP; (4) αPD‐1; (5) PAA‐CaS; (6) ZnPP@PAA‐CaS; (7) PAA‐CaS + αPD‐1; (8) ZnPP@PAA‐CaS + αPD‐1. Reproduced with permission.^[^ ^71^ ^]^ Copyright 2021, American Chemical Society.

CaS can undergo decomposition in an acidic environment, making it a promising nanomaterial for regulating Ca^2+^ homeostasis. The degradation products of CaS, namely Ca^2+^ and hydrogen sulphide (H_2_S), can serve as chemical messengers for intracellular transduction signals, and play an important role in regulating cellular activity. Thus, Liu et al. employed CaS to inhibit tumour growth through signalling transduction cascades (Figure 7A).^[^ ^71^ ^]^ The poly(acrylic acid) (PAA)‐stabilized CaS nanoparticles were utilized to construct nanomessengers, which were further loaded with zinc protoporphyrin (ZnPP) (Figure 7B) to amplify the messenger signal. Within acidic endosomes, the resulted ZnPP@PAA‐CaS nanomessengers gradually released chemical messengers Ca^2+^ and H_2_S to induce Ca^2+^ overload within tumour cells. Additionally, ZnPP produced ROS to further inhibit the cellular defence mechanism of the endogenous cytoprotective enzyme HO‐1. The restoration of inhibited signalling pathways subsequently triggered a series of signal transduction cascades that induced Ca^2+^‐overloaded tumour cell death, resulting in the release of tumour‐associated antigens.^[^ ^72^ ^]^ These antigens could activate anti‐tumour immunity and establish immune memory to prevent tumour metastasis and recurrence (Figure 7C–E). Therefore, this chemical messenger‐based nanoplatform amplified the regulation of cell behaviour mediated by chemical messengers (Ca^2+^, H_2_S) through cascade engineering to treat tumours and mediate immunotherapy.

##### CaF_2_

**FIGURE 8:**
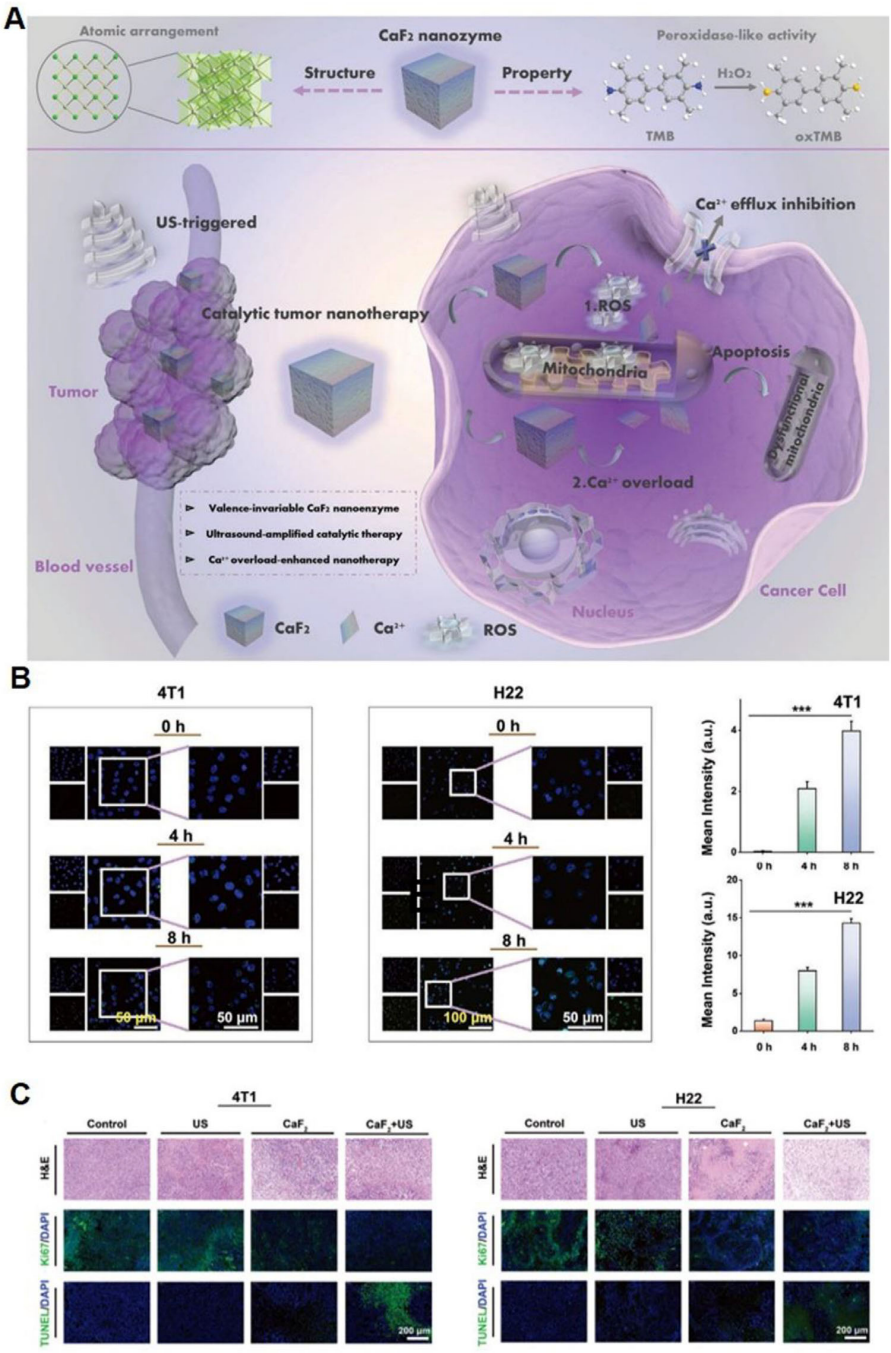
CaF_2_‐based nanoplatform for Ca^2+^ overload. (A) Schematic illustration for the underlying mechanism of US‐amplified CaF_2_ nanozyme for Ca^2+^‐overload‐assisted catalytic tumour therapy. (B) Intracellular Ca^2+^ ions in 4T1 cancer cells and H22 cancer cells. (C) H&E, Ki‐67, and TUNEL staining images of 4T1‐tumour and H22‐tumour sections from the tumour‐bearing mice in different treatment groups. Reproduced with permission.^[^ ^76^ ^]^ Copyright 2019, John Wiley & Sons.

The CaF_2_ nanosystem has been widely recognized as an exceptional optical substrate for diagnosis and cancer therapy because of its high biocompatibility, idiographic chemical stability and optical light transmission feature.^[^ ^73^, ^74^ ^]^ Recently, Li and co‐workers discovered that CaF_2_ nanocrystals exhibited peroxidase (POD)‐mimicking activity by generating ROS such as hydroxyl radicals (•OH), derived from the facet and crystal structure.^[^ ^75^ ^]^ Building on this discovery, Chang et al. demonstrated that ultrasound (US) could amplify the POD‐mimicking properties of CaF_2_ nanocrystals to produce ROS (Figure 8A).^[^ ^76^ ^]^ Combined with the release of exogenous Ca^2+^ from CaF_2_ nanocrystals, this system could prompt intracellular Ca^2+^ accumulation to induce mitochondrial dysfunction and apoptosis in breast (4T1) and hepatic carcinoma (H22) tumours both in vitro and in vivo (Figure 8B and C). This indicates that the CaF_2_ nanosystem holds significant potential in cancer therapy by its capacity to generate ROS and trigger Ca^2+^ overload, which can disrupt mitochondrial function and induce apoptosis in cancer cells.

##### Other forms of Ca^2+^‐based nanomaterials

**FIGURE 9:**
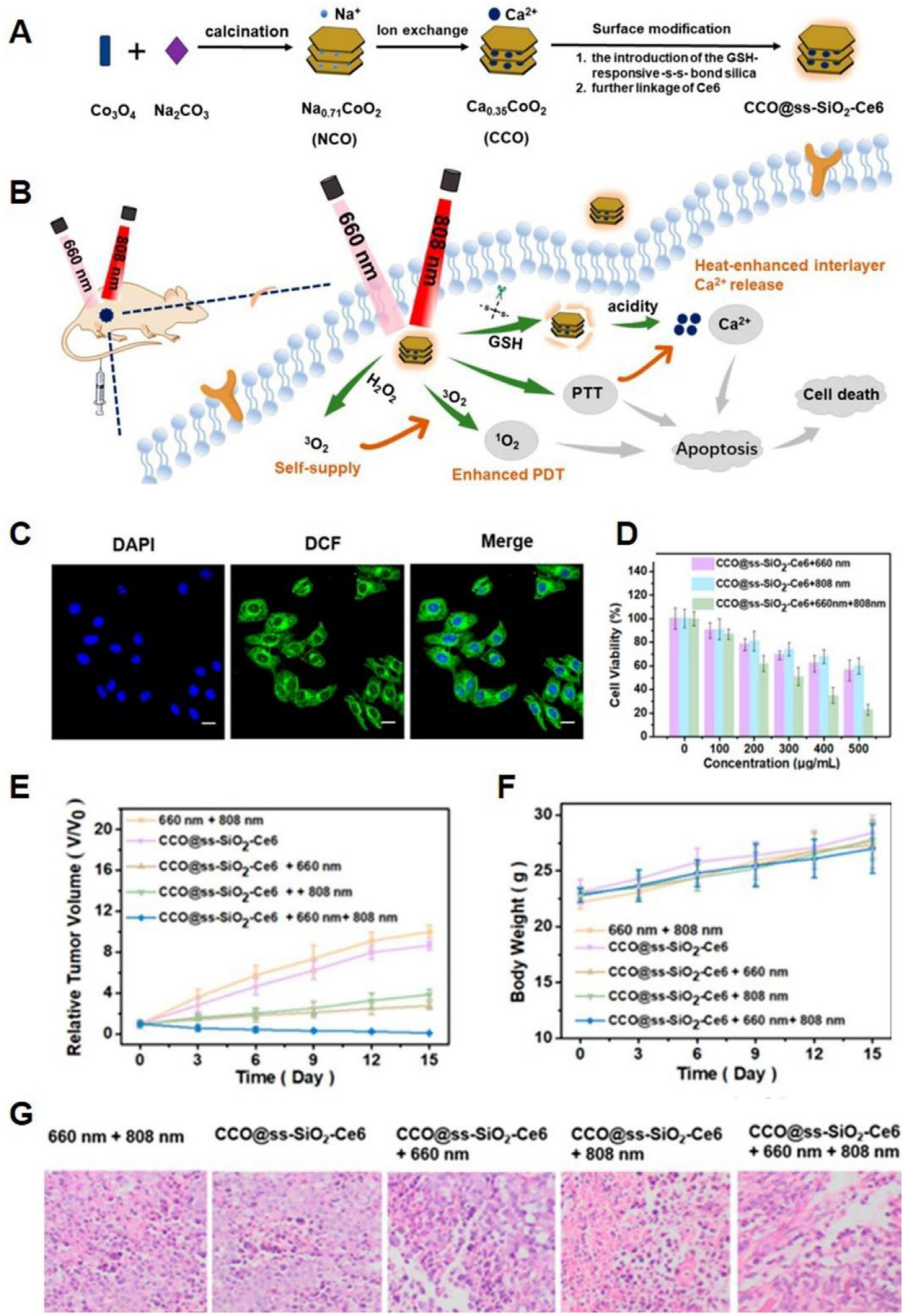
Other forms of Ca^2+^‐based nanomaterials for Ca^2+^ overload (CaxCoO_2_). (A) Schematic diagram of the synthesis procedure of CCO@ss‐SiO_2_‐Ce6 and (B) tumour therapy of synergistic induction of CCO@ss‐SiO_2_‐Ce6 heat‐enhanced Ca^2+^ overload and PDT/PTT. (C) Intracellular ROS generation in HepG2 cells treated with CCO@ss‐SiO_2_‐Ce6 under 660 nm laser irradiation. (D) Cell viability of HepG2 cells after incubated with different concentrations of CCO@ss‐SiO_2_‐Ce6. (E) Tumour volume, (F) body weight, and (G) H&E staining images of tumour slices from mice treated with various treatments after 15 days. Reproduced with permission.^[^ ^77^ ^]^ Copyright 2021, American Chemical Society.

Aside from the direct use of Ca^2+^‐based nanomaterials to induce Ca^2+^ overload, certain nanomaterials can load Ca^2+^ and disrupt intracellular Ca^2+^ homeostasis to trigger cancer cell death. Especially, the unique layered structure of CaxCoO_2_ (CCO) provides active vacancies that allow M ions (M = alkali metal or alkaline earth metals) to escape from the interlaminar layer, making it a potential candidate as a Ca^2+^ nanocarrier. Chen et al. developed a nanoagent with a layered structure that linked chlorin e6 (Ce6) photosensitizer (CCO@ss‐SiO_2_‐Ce6) to release Ca^2+^ between layers to induce Ca^2+^ overload and enhance PTT and PDT therapies under 808 and 660 nm laser irradiation (Figure 9A,B).^[^ ^77^ ^]^ Elevated temperatures also promoted the release of Ca^2+^. Moreover, CCO@ss‐SiO_2_‐Ce6 could catalyze the decomposition of intracellular H_2_O_2_ into O_2_ to alleviate hypoxia, promoting the production of singlet oxygen via Ce6 photosensitizer under 660 nm laser irradiation (Figure 9C). Finally, with synergetic Ca^2+^ release capacity as well as PTT and PDT effects, CCO@ss‐SiO_2_‐Ce6 exhibited significant tumour elimination ability both in vitro and in vivo (Figure 9D–G). This nanoagent opens up a new path for the development of emerging Ca^2+^‐overloaded nano agents in tumour therapy.

**FIGURE 10:**
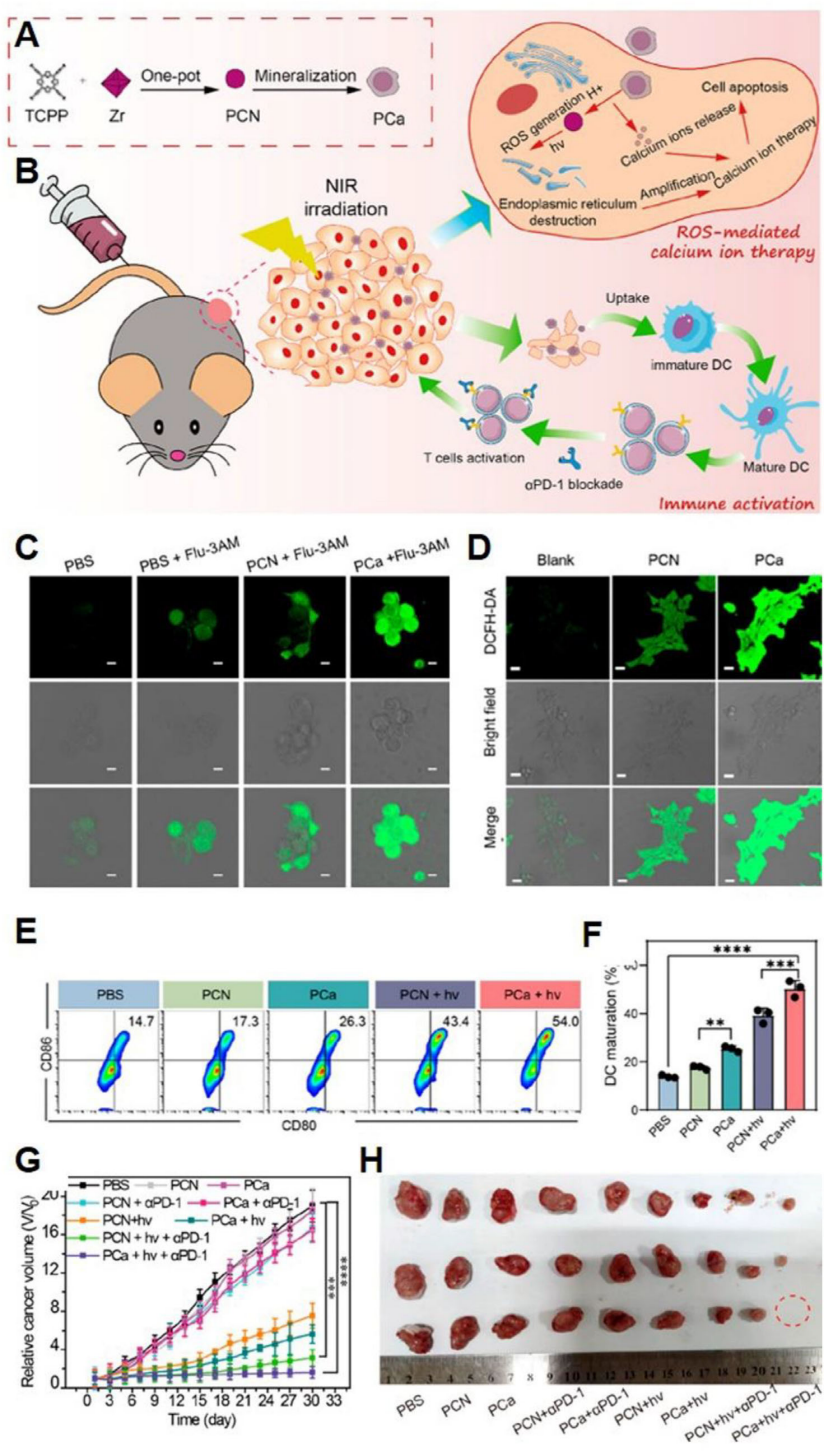
Other forms of Ca^2+^‐based nanomaterials for Ca^2+^ overload (Ca^2+^‐based MOF). (A) Schematic illustration for the synthesis process of PCa and (B) mechanism of PCa for amplified Ca^2+^ overload and activated immunotherapy. Fluorescent images to detect the (C) intracellular Ca^2+^ concentration and (D) ROS generation. (E, F) BMDC maturation analysis by flow cytometry. (G) Tumour growth curves and (H) photographs of collected tumours at the end of treatment after various treatments. Reproduced with permission.^[^ ^78^ ^]^ Copyright 2023, American Chemical Society.

Recently, metal‐organic framework (MOF) has gained considerable attention in biomedical applications, offering exciting prospects for the development of novel Ca^2+^‐based MOFs for expanded Ca^2+^ overload therapy. By exploiting this potential, Yu et al. developed mineralized porphyrin MOF (PCa) (Figure 10A) capable of achieving PDT and Ca^2+^ overload for enhanced therapeutic outcomes.^[^ ^78^ ^]^ The generated ROS after PDT could destroy cell calcium buffering capacity, amplifying the cell damage caused by Ca^2+^ overload (Figure 10B). Treatment of 4T1 cells with PCa and subsequent 630 nm irradiation showed increased intracellular Ca^2+^ concentration (Figure 10C) and elevated oxidative stress levels (Figure 10D). Furthermore, they could effectively enhance the maturation of bone marrow‐derived dendritic cells (BMDC) (Figure 10E,F), which could achieve obvious antitumour activity (Figure 10G,H).

#### 2.1.2 Non‐calcium nanomaterials indirectly trigger Ca^2+^ overload

##### Plasma membrane damage to trigger Ca^2+^ overload

**FIGURE 11:**
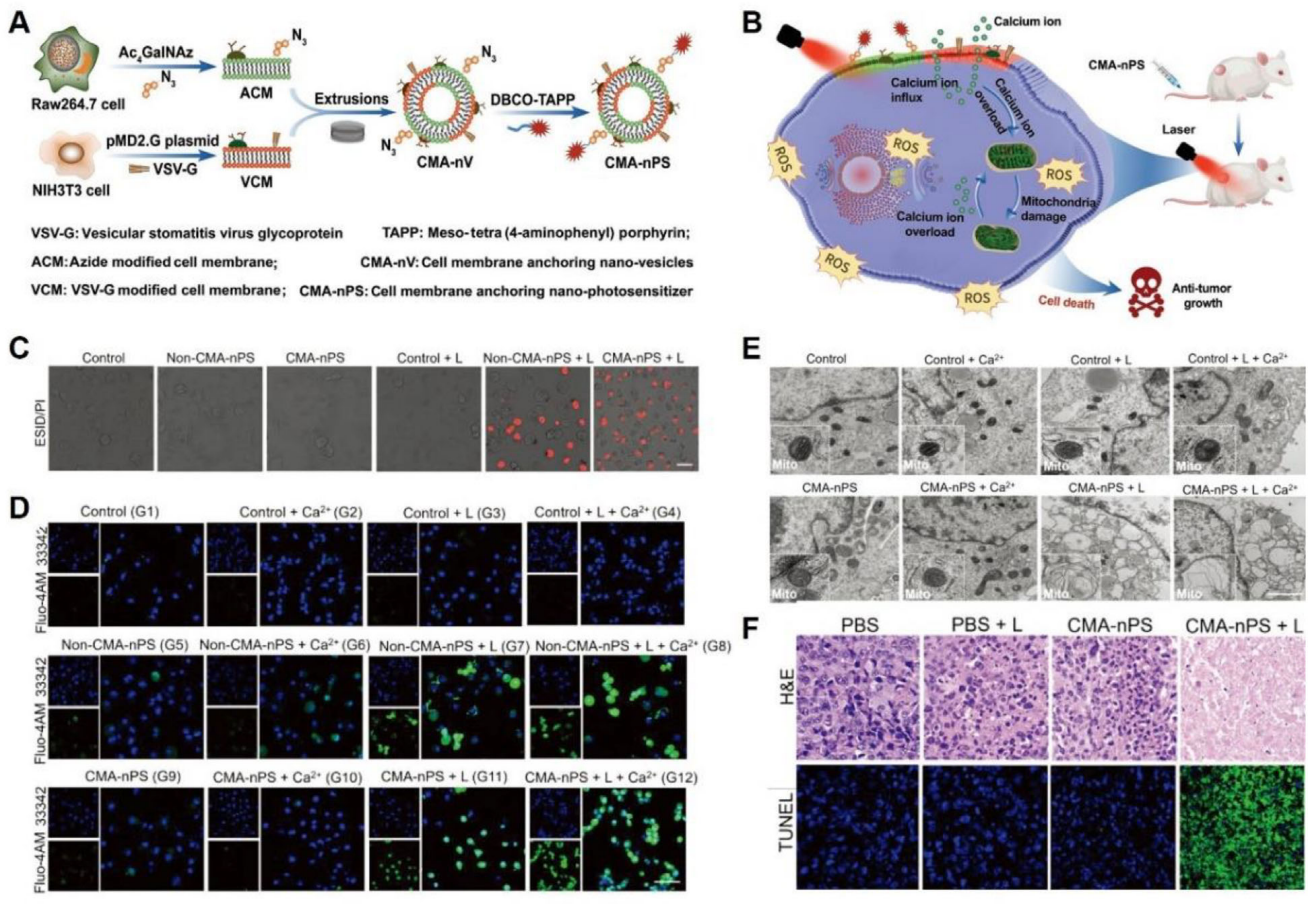
Plasma membrane damage to trigger Ca^2+^ overload. (A) Schematic diagram of the preparation process of CMA‐nPS and (B) their capacity to generate abundant ROS and introduce intracellular Ca^2+^ overload for enhanced antitumour efficiency. (C) Fluorescent images to detect cell membrane integrity and (D) intracellular Ca^2+^ concentration. (E) TEM images of H1299 cells after different treatments to observe mitochondrial integrity. (F) H&E and TUNEL staining images of H1299 tumour‐bearing mice after different treatments. Reproduced with permission.^[^ ^79^ ^]^ Copyright 2022, John Wiley & Sons.

Recent studies suggest that the damage to the plasma membrane could trigger an influx of extracellular Ca^2+^, leading to intracellular Ca^2+^ overload and subsequent cell death. He et al. developed an approach for tumour‐specific Ca^2+^ overload therapy and PDT by constructing a cell membrane‐anchored nano‐photosensitizer (CMA‐nPS) (Figure 11A).^[^ ^79^ ^]^ Through the targeting ability of two types of functionalized cell membranes, CMA‐nPS was able to primarily anchor to the membrane of lung cancer cells and generate local ROS under laser irradiation (Figure 11B). This process directly damaged the lung cancer cell membrane (Figure 11C), causing Ca^2+^ influx (Figure 11D), disrupting Ca^2+^‐buffering capacity, leading to mitochondrial dysfunction (Figure 11E), and accelerating cell death (Figure 11F). This strategy offers a promising new synergistic strategy for indirect Ca^2+^‐overload‐based cancer therapy.

##### Nitric oxide (NO) to trigger Ca^2+^ overload

**FIGURE 12:**
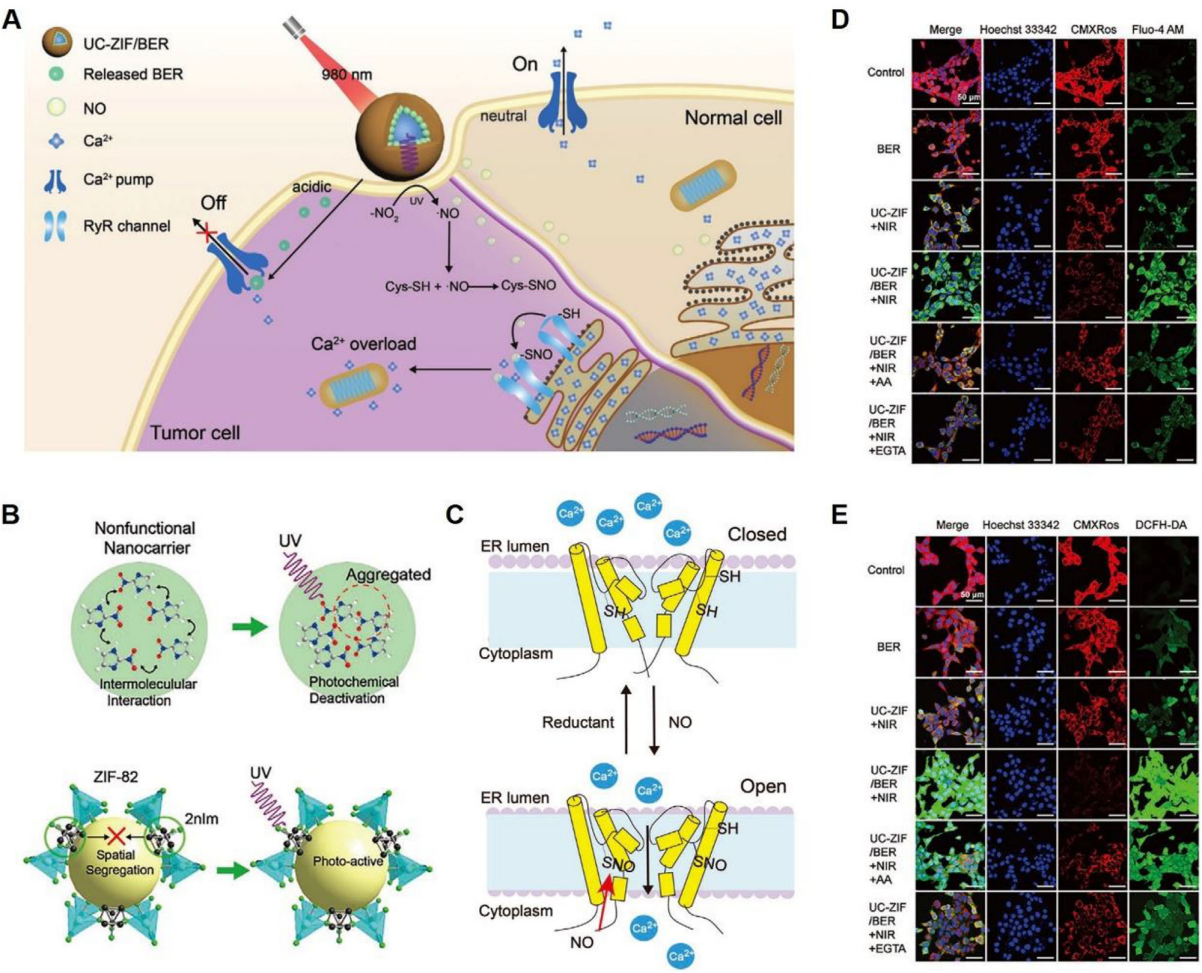
NO to trigger Ca^2+^ overload. (A) Schematic illustration of intracellular calcium stores modulated by NO for Ca^2+^ overload‐initiated cancer therapy. (B) Aggregation quenching effect of 2 nitroimidazole (2nIm) molecules (top). The photochemical activity of 2nIm could be improved by ZIF‐82 (low). (C) Mechanisms of NO activating RyRS. Fluorescent images to detect (D) intracellular Ca^2+^ concentration and (E) ROS generation. Reproduced with permission.^[^ ^82^ ^]^ Copyright 2021, John Wiley & Sons.

It has been reported that NO can activate the overexpressed RyR channels in cancer cells, resulting in abrupt Ca^2+^ release from ER and ultimately inducing cell apoptosis.^[^ ^80^, ^81^ ^]^ Building on this knowledge, Chu et al. developed a Ca^2+^ regulating system that utilized non‐toxic internal Ca^2+^ in cancer cells for Ca^2+^ release, which consisted of upconversion nanoparticles (UCNPs), nanosized zeolitic nitro‐/nitrile‐imidazole framework‐82 (ZIF‐82) (denoted as UC‐ZIF) and the loaded berberine (BER) (denoted as UC‐ZIF/BER). The system involved synchronous channel “ON” in calcium storage, Ca^2+^ pump “OFF” for inhibitory Ca^2+^ efflux, and rationally designed nanoparticles for NIR‐triggered NO generation and BER release.^[^ ^82^ ^]^ Upon NIR laser irradiation, the ultraviolet (UV) emission from UCNPs triggered BER release and NO production (Figure 12A,B). The lipophilic NO rapidly spread and invaded the overexpressed RyRs in breast cancer cells for protein S‐nitrosylation, which opened RyRs to allow Ca^2+^ flow out from the ER.^[^ ^83^ ^]^ Meanwhile, as a Ca^2+^ pump inhibitor, BER bound to Ca^2+^‐excreted pumps to inhibit the excretion of Ca^2+^ in the cell membrane, resulting in high intracellular Ca^2+^ levels (Figure 12C,D).^[^ ^84^, ^85^ ^]^ This Ca^2+^ overload disturbed cellular metabolism, causing an increase in ROS production (Figure 12E) and ultimately inducing apoptosis in cancer cells without systemic toxicity. This approach utilizes the endogenous Ca^2+^ signalling system of cells to selectively trigger tumour cell death, providing a novel strategy for cancer treatment that circumvents the use of exogenous cytotoxic drugs.

### 2.2 Ca^2+^ inhibition

Studies have demonstrated that the overexpression of transmembrane Ca^2+^ channels and the hyperactivity of Ca^2+^‐related proteins in tumour cells result in abnormally high intracellular Ca^2+^ concentrations, which are crucial for tumourigenesis and tumour progression. Moreover, these elevated levels of intracellular Ca^2+^ can promote tumour cell proliferation and migration and induce the expression of drug resistance‐related protein P‐glycoprotein (P‐gp). The mechanism allows tumour cells to withstand drug treatment and acquire resistance, resulting in poor treatment outcomes. Recently, nanoparticles have been designed to regulate Ca^2+^ signalling negatively by blocking Ca^2+^ influx, decreasing Ca^2+^ signalling, or directly capturing Ca^2+^. This strategy holds great potential in reversing tumour drug resistance and enhancing the therapeutic efficacy of drugs against drug‐resistant tumours.

#### 2.2.1 Ca^2+^ chelation agent capture of Ca^2+^

##### Phytic acid (PA) chelating

**FIGURE 13:**
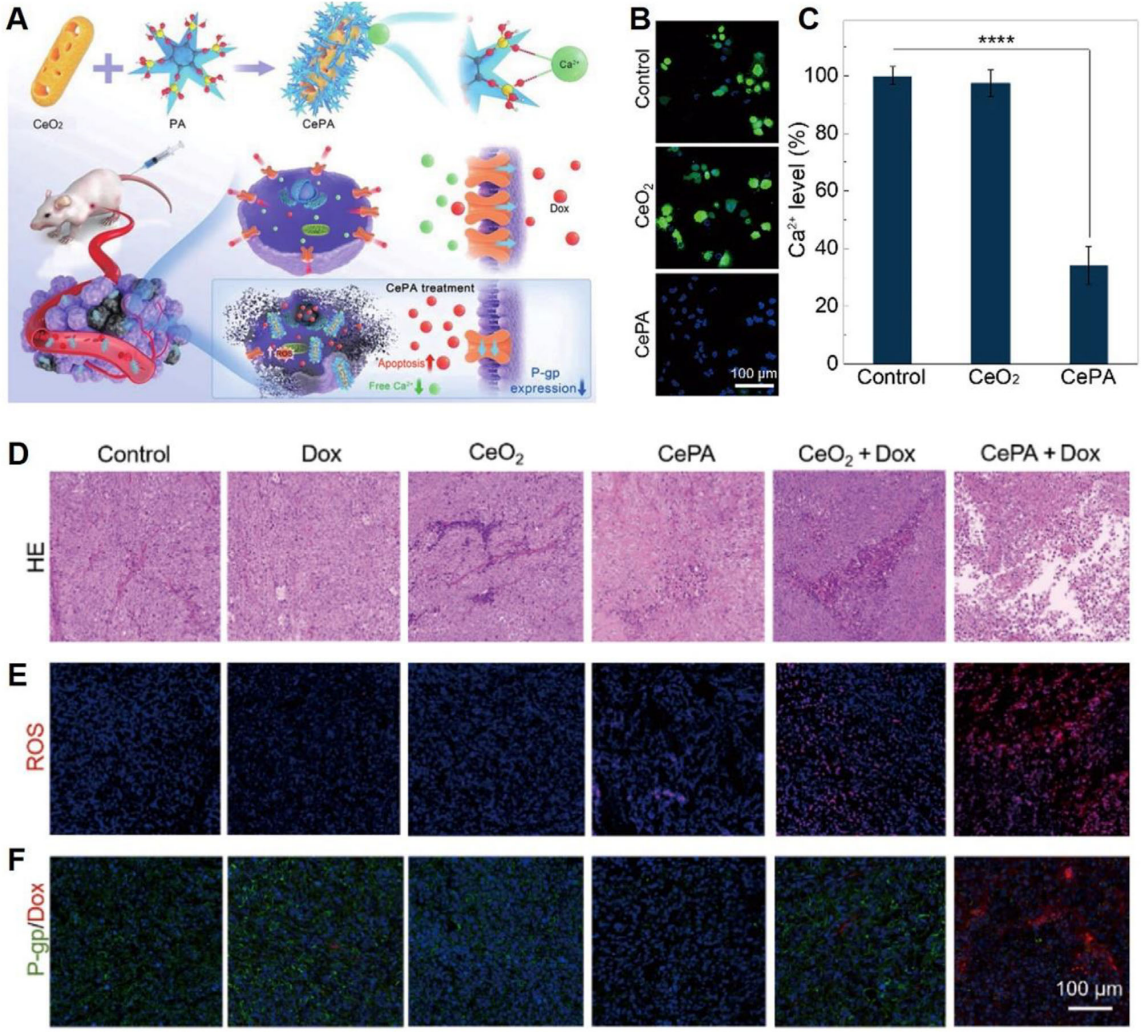
PA chelating to capture Ca^2+^ for Ca^2+^ inhibition. (A) Schematic illustration of the synthesis of CePA and their antitumour mechanism through PA‐chelated Ca^2+^ for overcoming drug resistance and chemotherapy. (B, C) Intracellular Ca^2+^ concentration after different treatments. (D) H&E staining and (E) immunofluorescence staining of ROS, (F) P‐gp and Dox. Reproduced with permission.^[^ ^90^ ^]^ Copyright 2022, Springer.

PA, also known as inositol hexaphosphoric acid, is an organic acid that can be extracted from various plant sources such as seeds, beans, cereals, vegetables, and fruits. It is an abundant, low‐cost, and environmentally friendly natural compound that offers numerous health benefits, such as acting as an antioxidant, anticancer agent, and chelating agent.^[^ ^86^, ^87^, ^88^ ^]^ Structurally, PA contains six phosphates and exhibits potent chelating activity against diverse metal cations like Mg^2+^ and Zn^2+^. Moreover, it has been approved by the Food and Drug Administration (FDA) in the US as a generally recognized safe (GRAS) material. Previous studies have demonstrated that oral administration of PA at a dose of up to 200 mg kg^−1^ does not induce any intestinal irritation in rats.^[^ ^89^ ^]^ In recent research, Tian et al. designed PA‐modified CeO_2_ (CePA) nano‐inhibitors as a safe and effective Ca^2+^ inhibitor to overcome drug resistance in tumours that overexpress intracellular Ca^2+^ (Figure 13A).^[^ ^90^ ^]^ CePA was found to effectively decrease excessive levels of free Ca^2+^ in cells (Figure 13B and C). In vivo experiments showed CePA+Dox could induce tumour cell apoptosis (Figure 13D), strengthened the ROS generation intratumour (Figure 13E), downregulated intratumoural P‐gp expression (green fluorescence) and enhanced intratumoural ROS level (red fluorescence) (Figure 13F), significantly enhance drug accumulation in drug‐resistant tumours. Furthermore, as a pH‐regulated nanozyme, nanoceria considerably alleviated the harmful effects of chemotherapeutic drugs on normal cells and organs, thereby enhancing the survival rate of mice. The co‐administration of Dox and CePA exhibited a remarkable enhancement in the efficacy of chemotherapy while concurrently mitigating its adverse effects. This strategy of negative regulation of intracellular Ca^2+^ presents an interesting approach for treating drug‐resistant tumours and has the potential to overcome the limitations of conventional chemotherapy drugs.

##### 2.2.1.2 Ethylene glycol bis (2‐aminoethyl ether)‐*N,N,N′,N*′‐tetra‐acetic acid (EGTA) chelating

**FIGURE 14:**
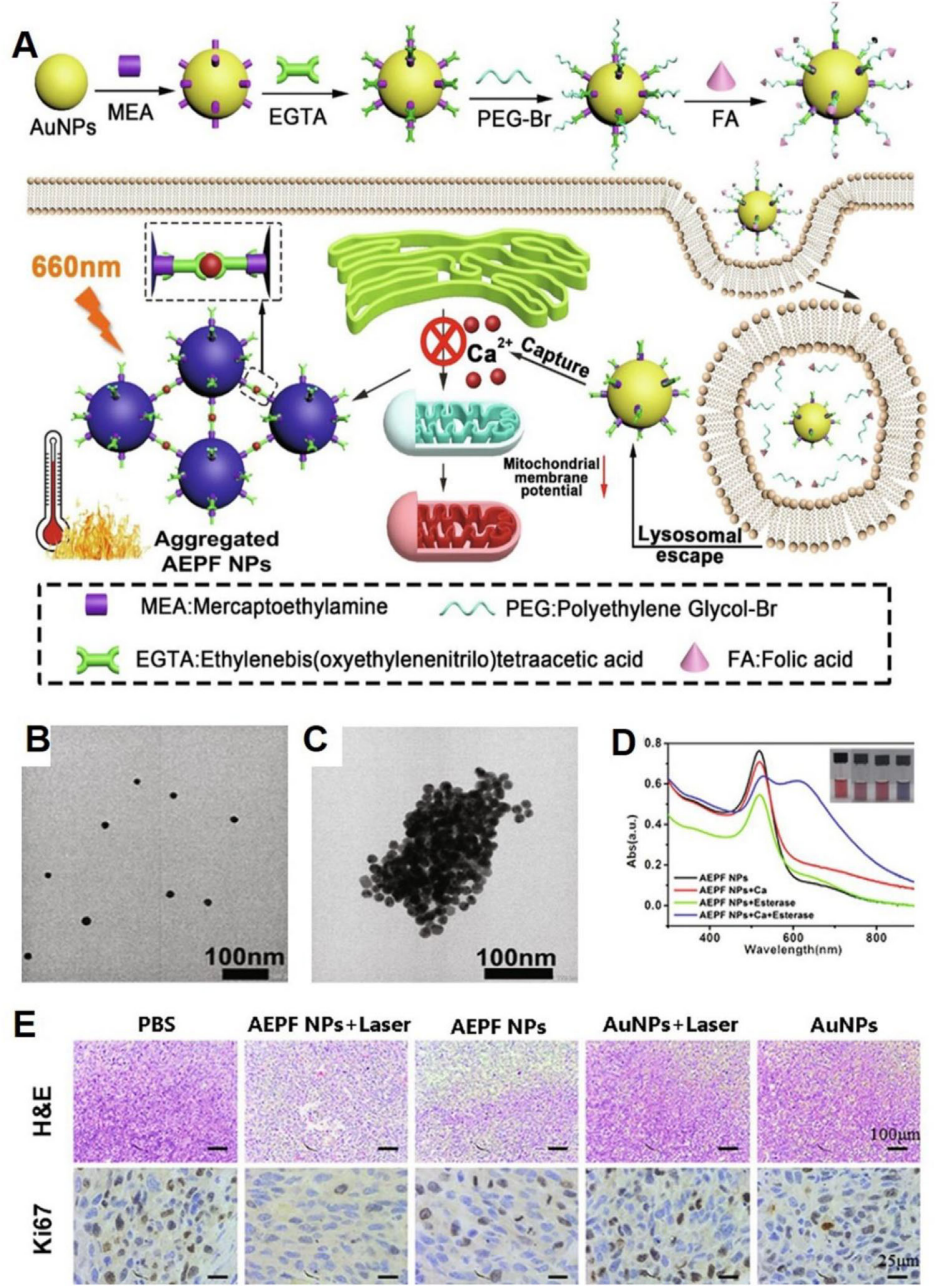
EGTA chelating to capture Ca^2+^ for Ca^2+^ inhibition. (A) Schematic illustration of the synthesis of AEPF NPs and their antitumour performance via EGTA‐captured Ca^2+^ and photothermal therapy. (B) TEM images of AEPF NPs in Ca^2+^ solution before adding esterase. (C) TEM images of AEPF NPs in Ca^2+^ solution after adding esterase. (D) UV–vis spectrum and corresponding photographs of AEPF NPs solution with and without Ca^2+^ and esterase. (E) H&E and Ki‐67 staining images of tumour sections in different treatment groups. Reproduced with permission.^[^ ^94^ ^]^ Copyright 2021, Elsevier.

As a typical Ca^2+^ chelating agent, EGTA can capture Ca^2+^ effectively in tumour cells, thus regulating Ca^2+^ concentration.^[^ ^91^, ^92^, ^93^ ^]^ Guo and colleagues synthesized FA‐PEG‐EGTA‐AuNPs nanoparticles (AEPF NPs) by co‐modifying gold nanoparticles (Au NPs) with EGTA and folic acid (FA), and connecting them with polyethylene glycol 4000. These nanoparticles were designed to regulate intracellular Ca^2+^ levels and trigger PTT for synergistic tumour treatment (Figure 14A).^[^ ^94^ ^]^ AEPF NPs maintained a stable “off state” during blood circulation post‐injection due to the conjugation of Au‐EGTA with FA‐PEG via an ester bond, which deactivated the nanoparticles and prevented unexpected interference from systemic Ca^2+^ and non‐specific protein adsorption before reaching tumour tissue. Following FA‐mediated tumour accumulation^[^ ^95^ ^]^ and endocytosis,^[^ ^96^ ^]^ PEG‐FA could be removed by over‐expressed esterase in tumour cells, recovering the Ca^2+^ chelating functions of EGTA to truncate Ca^2+^ signalling for tumour inhibition. Moreover, the chelation of Ca^2+^ would trigger intracellular aggregation of small Au nanoparticles (Figure 14B and C), leading to a shift in their absorbance peak from 500 to 660 nm (Figure 14D) and enabling superior PTT upon irradiation at 660 nm, thereby enhancing tumour treatment. This approach not only took advantage of the tumour‐specific environment (abnormally high Ca^2+^ concentration) to achieve precise targeting, but also modified the tumourigenic environment by Ca^2+^ regulation to enhance therapeutic efficacy, thereby offering a promising strategy for tumour treatment development (Figure 14E).

#### 2.2.2 Knockdown of T‐type Ca^2+^ channel

**FIGURE 15:**
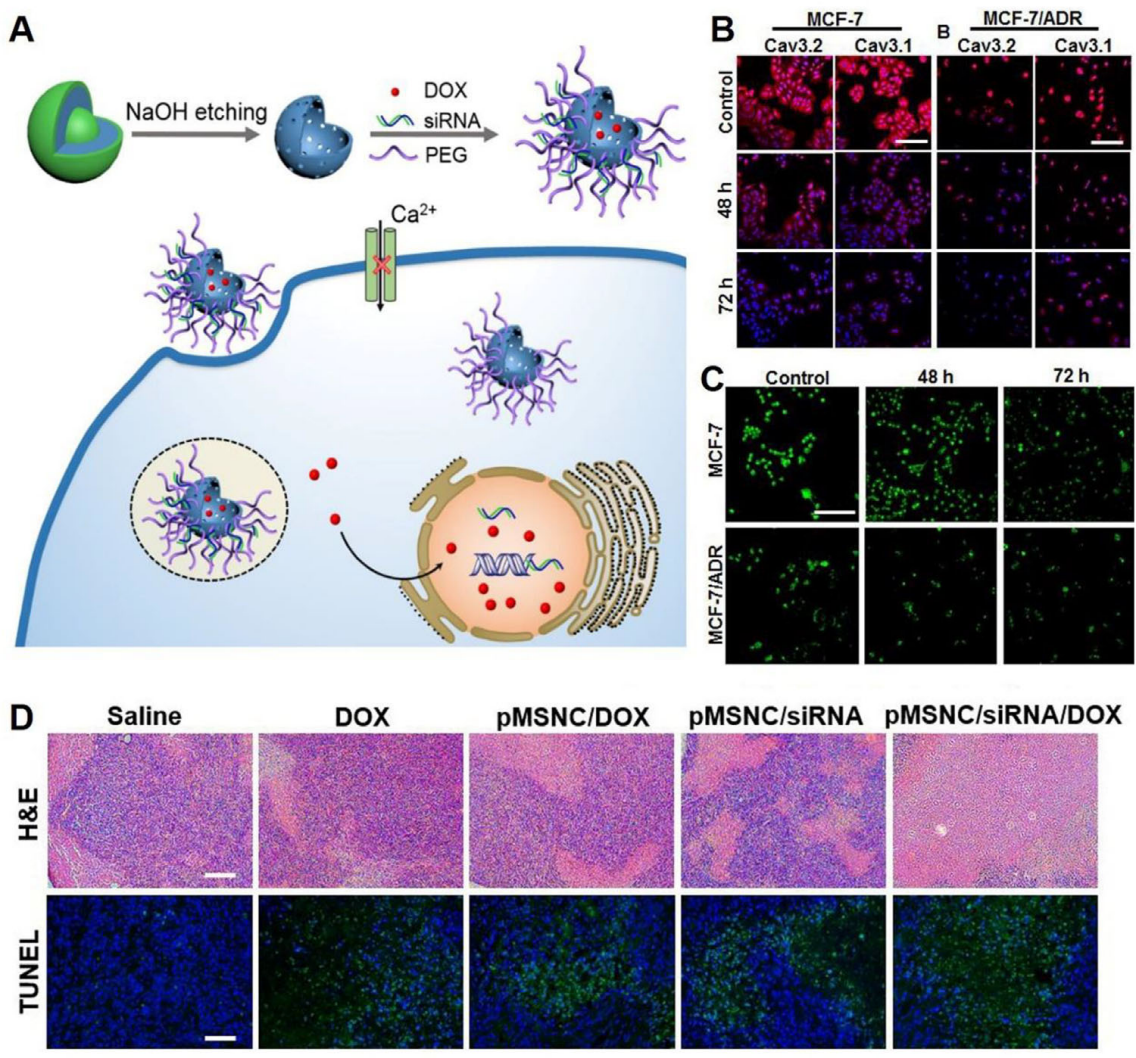
Knockdown of T‐type Ca^2+^ channel for Ca^2+^ inhibition. (A) Schematic illustration of the synthesis process of MSNCs and their application in tumour therapy through downregulation of T‐type Ca^2+^ channel expression, reduction in intracellular Ca^2+^ concentration, and chemotherapy. Fluorescent images to detect (B) Ca_v_ 3.1 and Ca_v_ 3.2 T‐type Ca^2+^ channels and (C) intracellular Ca^2+^ concentration. (D) H&E and TUNEL staining images of tumour sections after different treatments. Reproduced with permission.^[^ ^23^ ^]^ Copyright 2019, American Chemical Society.

Several studies have demonstrated that blocking the T‐type Ca^2+^ channel using antagonists^[^ ^22^, ^24^ ^]^ or siRNA^[^ ^24^ ^]^ could enhance the sensitivity of cancer cells to cytotoxic drugs in vitro. Nonetheless, the employment of antagonists and siRNA is hindered by their high toxicity and instability in vivo, which present significant challenges to their practicality and may result in undesirable side effects. To overcome these limitations and enhance specificity and stability, Wang et al. fabricated silica nanocapsules (MSNCs) with a mesoporous and hollow structure as a drug carrier for co‐delivery of T‐type Ca^2+^ channel siRNA and DOX (Figure 15A).^[^ ^23^ ^]^ This co‐loading system exhibited high drug‐loading efficiency and potent treatment efficacy against drug‐resistant breast cancer. The synergistic therapeutic effect on MCF‐7/ADR cells was attributed to the knockdown of the overexpressed T‐type Ca^2+^ channel (Figure 15B), which reduced cytosolic Ca^2+^ concentration (Figure 15C), induced G0/G1 phase cell cycle arrest, and enhanced intracellular drug accumulation. Finally, this system demonstrated favourable effects on drug‐resistant breast cancer, resulting in a tumour suppression rate that increased by over 46% compared to drugs alone (Figure 15D). These findings offer novel targets and approaches for drug and gene delivery to drug‐resistant tumours.

## 3 NANOPLATFORMS MODULATING CA^2+^ HOMEOSTASIS IN TARGETING VARIOUS CELLS

After introducing various strategies for Ca^2+^ regulation, our next focus is on studying Ca^2+^ regulation for cancer treatment targeting different cells. In addition to the methods mentioned above for directly regulating tumour cell death in cancer treatment (which will not be reiterated in this section), Ca‐based nanoplatforms can also participate in tumour immune response, by directly or directly regulating Ca^2+^ of immune cells. Increasing evidence suggests that Ca^2+^ plays a crucial role in immune cell activation and phenotypic modulation. Specifically, Ca‐based nanoplatforms have the potential to target cancer cells and induce ICD to generate antigens.^[^ ^29^ ^]^ They can also regulate immune cells, such as dendritic cells (DCs) to enhance antigen presentation,^[^ ^30^ ^]^ T cells to boost immune responses^[^ ^27^ ^]^ as well as macrophages to amplify anticancer effects.^[^ ^28^ ^]^

### 3.1 Ca^2+^ inducing ICD of cancer cells to produce tumour‐associated antigens

**FIGURE 16:**
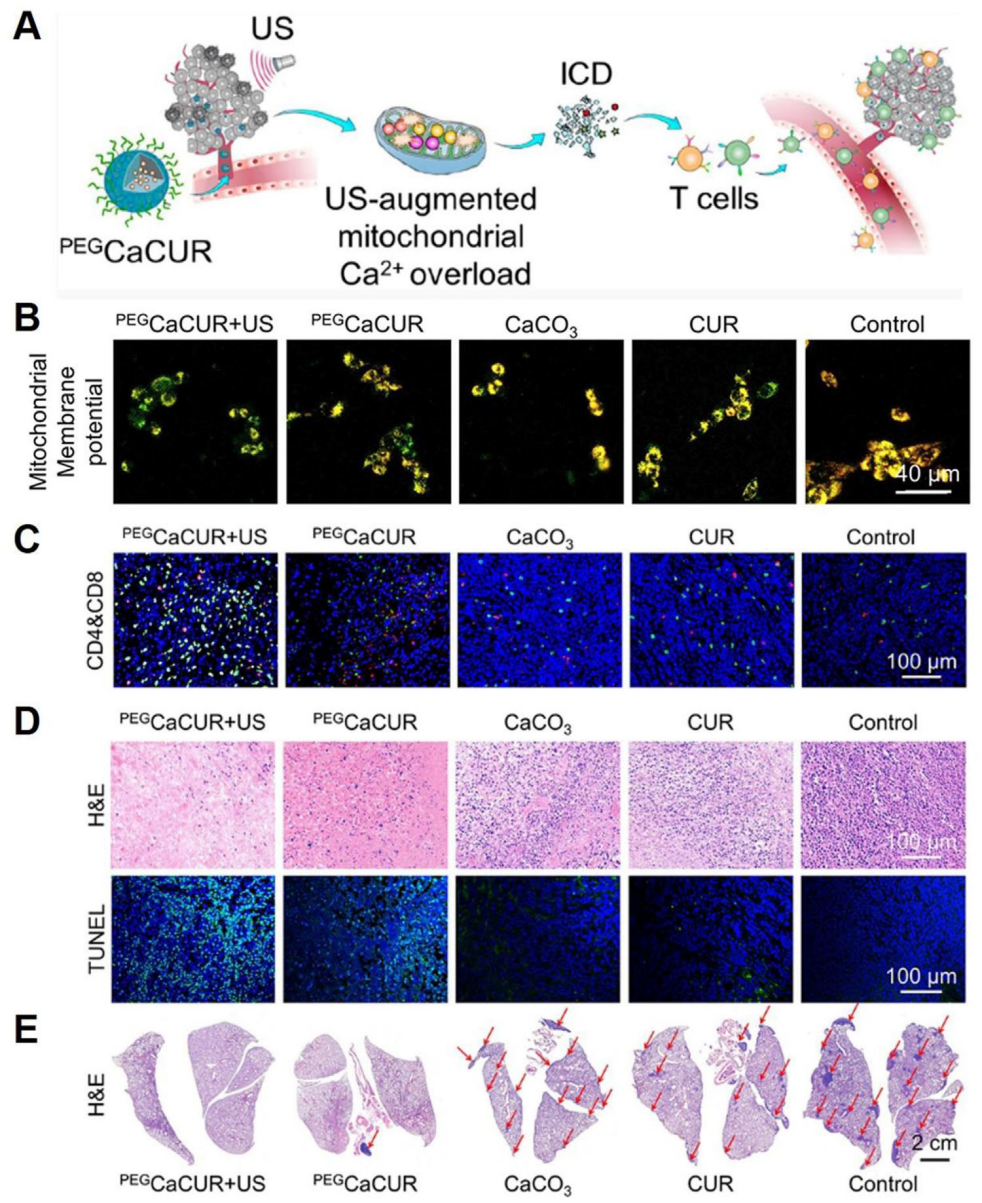
Ca^2+^ inducing ICD of cancer cells to produce tumor‐associated antigens. (A) Schematic illustration of US‐augmented collaborative Ca^2+^ overload and immunotherapy utilizing PEGCaCUR. (B) Fluorescent images to detect mitochondrial membrane potential. (C) Immunofluorescence staining of intratumoural CD4 (red fluorescence) and CD8 (green fluorescence) T cells after different treatments. (D) H&E and TUNEL staining images of tumour sections after different treatments. (E) H&E‐staining of lung sections at the end of different treatments. Tumour metastasis was indicated by red arrows. Reproduced with permission.^[^ ^29^ ^]^ Copyright 2021, American Chemical Society.

ICD plays a pivotal role in the tumour immune cycle by inducing the generation of multiple tumour‐associated antigens (TAAs), and has been a focal point of immunotherapy for many years.^[^ ^97^ ^]^ ICD inducers elicit damage‐associated molecular pattern (DAMP) signalling in the treated tumour, resulting in the translocation of calreticulin to the cell surface, the secretion of adenosine triphosphate (ATP), and the passive secretion of high mobility group protein B1 (HMGB1).^[^ ^98^, ^99^ ^]^ Although various ICD inducers have been reported, such as chemotherapeutic drugs^[^ ^100^ ^]^ and photosensitizers,^[^ ^101^ ^]^ they may present serious side effects with limited ICD induction efficiency, requiring relatively high doses and frequent dosing, and a lack of selectivity for cancer cells.^[^ ^102^, ^103^ ^]^ Recently, Ca^2+^ has emerged as a promising inducer of ICD due to its high efficacy and biosafety. Because Ca^2+^ is mainly stored in mitochondria and ER, the disruption of mitochondrial Ca^2+^ homeostasis could precisely regulate ROS generation, which would stimulate DAMPs to trigger ICD and initiate defensive anti‐tumour immunity. To harness its potential, Zheng et al. developed a multifunctional Ca^2+^ nanomodulator by combining acid‐sensitive PEG‐modified CaCO_3_ with CUR in a simple one‐pot method (PEGCaCUR) (Figure 16A).^[^ ^29^ ^]^ Upon exposure to acidic conditions, a significant amount of Ca^2+^ was released, leading to an increase in intracellular Ca^2+^ levels. Furthermore, the liberated CUR facilitated Ca^2+^ release from the endoplasmic reticulum into the cytoplasm while inhibiting Ca^2+^ efflux and inducing mitochondrial dysfunction (Figure 16B) through Ca^2+^ overload, which could be further augmented by the US. Moreover, PEGCaCUR was found to induce ICD (Figure 16C) because of mitochondrial Ca^2+^ overload to effectively suppress tumour growth and metastasis (Figure 16D,E).

### 3.2 Ca^2+^ mediating autophagy of DCs to promote the presentation of antigens

**FIGURE 17:**
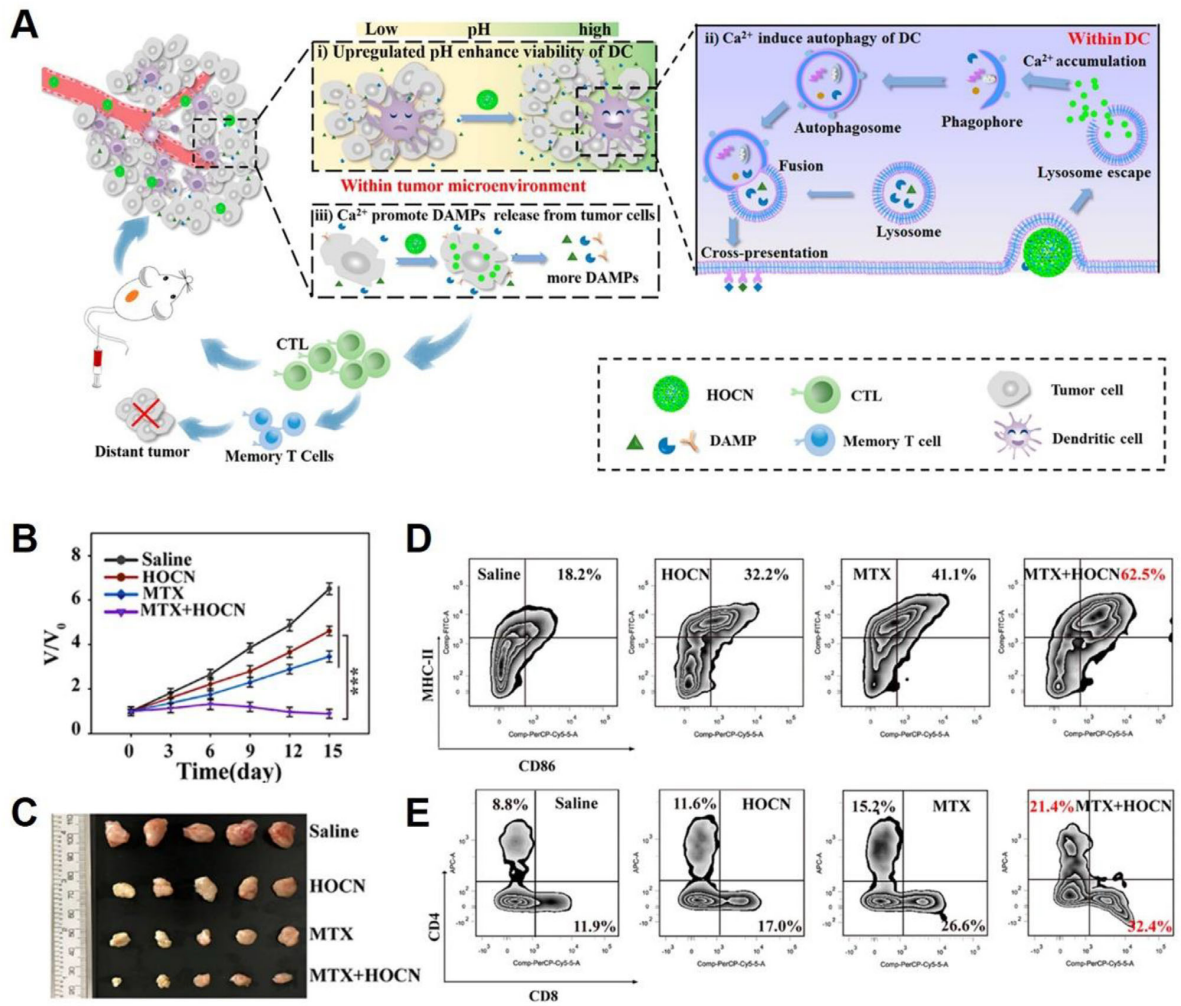
Ca^2+^ mediating autophagy of DCs to promote the presentation of antigens. (A) Schematic illustration of HOCN overcoming multiple barriers in DCs’ antigen cross‐presentation for chemo‐immunotherapy by neutralizing tumour acidity, inducing autophagy in DCs, and promoting Ca^2+^ overload to enhance DAMPs released from tumour cells. (B) Tumour growth curves and (C) representative tumour tissues from each group after different treatments. (D) DC maturation rates and (E) CD^8+^ and CD^4+^ T lymphocytes were examined by flow cytometry. Reproduced with permission.^[^ ^30^ ^]^ Copyright 2020, American Chemical Society.

Ca^2+^ also plays a crucial role in the autophagy process, whereby damaged organelles or proteins are encapsulated in autophagy vacuoles and transferred to lysosomes for degradation and recycling.^[^ ^104^ ^]^ Autophagy facilitates the digestion and processing of antigens during DCs antigen presentation.^[^ ^105^, ^106^ ^]^ However, the autophagy ability of DCs is inhibited in the TME, resulting in inefficient antigen processing,^[^ ^107^ ^]^ which may restrict the efficacy of cancer chemotherapy and immunization due to the insufficient antigen presentation capacity of DCs.^[^ ^108^ ^]^ Therefore, regulating Ca^2+^ levels in DCs has the potential to improve autophagy and thus enhance immunotherapy.^[^ ^109^ ^]^ An et al. developed a Ca^2+^ nanogenerator, named honeycomb OVA@CaCO_3_ nanoparticles (HOCN), to improve the immune responses induced by chemotherapy (Figure 17A).^[^ ^30^ ^]^ The HOCN was designed to provide an effective therapeutic effect (Figure 17B,C) by overcoming the barriers in DCs antigen cross‐presentation by augmenting Ca^2+^ production and disrupting the autophagy inhibitory conditions of DCs. Additionally, the HOCN mitigated tumour acidity and revitalized DCs, thereby contributing to improved immune response (Figure 17D,E).

### 3.3 Ca^2+^ functioning as a vaccine adjuvant to enhance T cell responses

**FIGURE 18:**
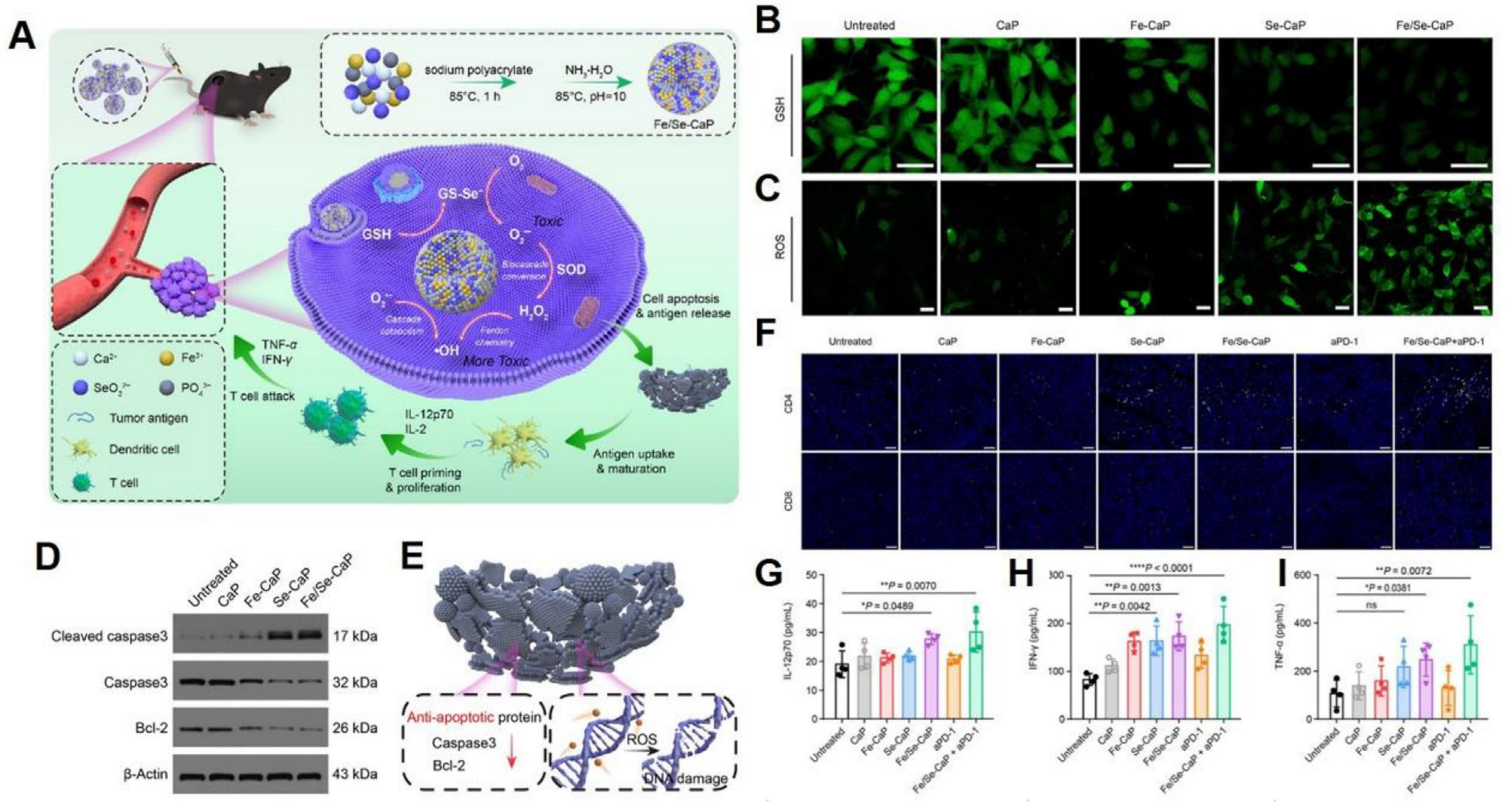
Ca^2+^ functioning as a vaccine adjuvant to enhance T cell responses. (A) Schematic illustration of the fabrication of Fe/Se‐CaP nanohybrid and their antitumour mechanisms involving GSH depletion, ROS generation, and vaccine adjuvant functions. Fluorescent images to detect (B) intracellular GSH level and (C) ROS generation. (D) Western blotting of apoptosis‐associated proteins. (E) Schematic illustration of the cell apoptosis mechanism. (F) Immunofluorescence staining of intratumoural CD4 (yellow fluorescence) and CD8 (red fluorescence) T cells after different treatments. Intratumoural IL‐12p70 (G), IFN‐γ (H), and TNF‐α (I) expression examined by enzyme‐linked immunosorbent assay (ELISA). Reproduced with permission.^[^ ^113^ ^]^ Copyright 2022, Elsevier.

In the development of vaccines, the selection of appropriate adjuvants is a crucial step. Among various nanoparticles developed as immune adjuvants,^[^ ^110^, ^111^ ^]^ CaP has demonstrated significant potential and has undergone extensive research.^[^ ^112^, ^113^ ^]^ Extensive studies have demonstrated that Ca‐based nanomaterials could avoid lysosomal degradation and promote cross‐presentation of antigens to exert the therapeutic action of CTLs. Especially, CaP, when used as an adjuvant, can stimulate the immune response of helper T cells^[^ ^27^, ^114^ ^]^ and activate NOD‐like receptor protein 3 (NLRP‐3) inflammasomes, thereby promoting the production of cytokines such as IL‐1β and facilitate T cell responses.^[^ ^115^, ^116^ ^]^ Recently, Peng et al. constructed a ferric ion and selenite‐codoped calcium phosphate (Fe/Se‐CaP) nanohybrid (Figure 18A) to catabolize endogenous GSH (Figure 18B), and generate abundant ROS (Figure 18C) for tumour cells apoptosis (Figure 18D,E).^[^ ^113^ ^]^ Wherein, CaP acted as a vaccine adjuvant and was beneficial to the proliferation of DCs, the increase of CD^4+^/CD^8+^ T cells (Figure 18F), the production of interleukin‐12p70 (Figure 18G), interferon‐γ (IFN‐γ) (Figure 18H) and tumour necrosis factor‐α (TNF‐α) (Figure 18I). The Fe/Se‐CaP system effectively activated the adaptive immune response and induced a robust immune response for tumour inhibition.

### 3.4 Ca^2+^ regulation of macrophage M1 polarization

**FIGURE 19:**
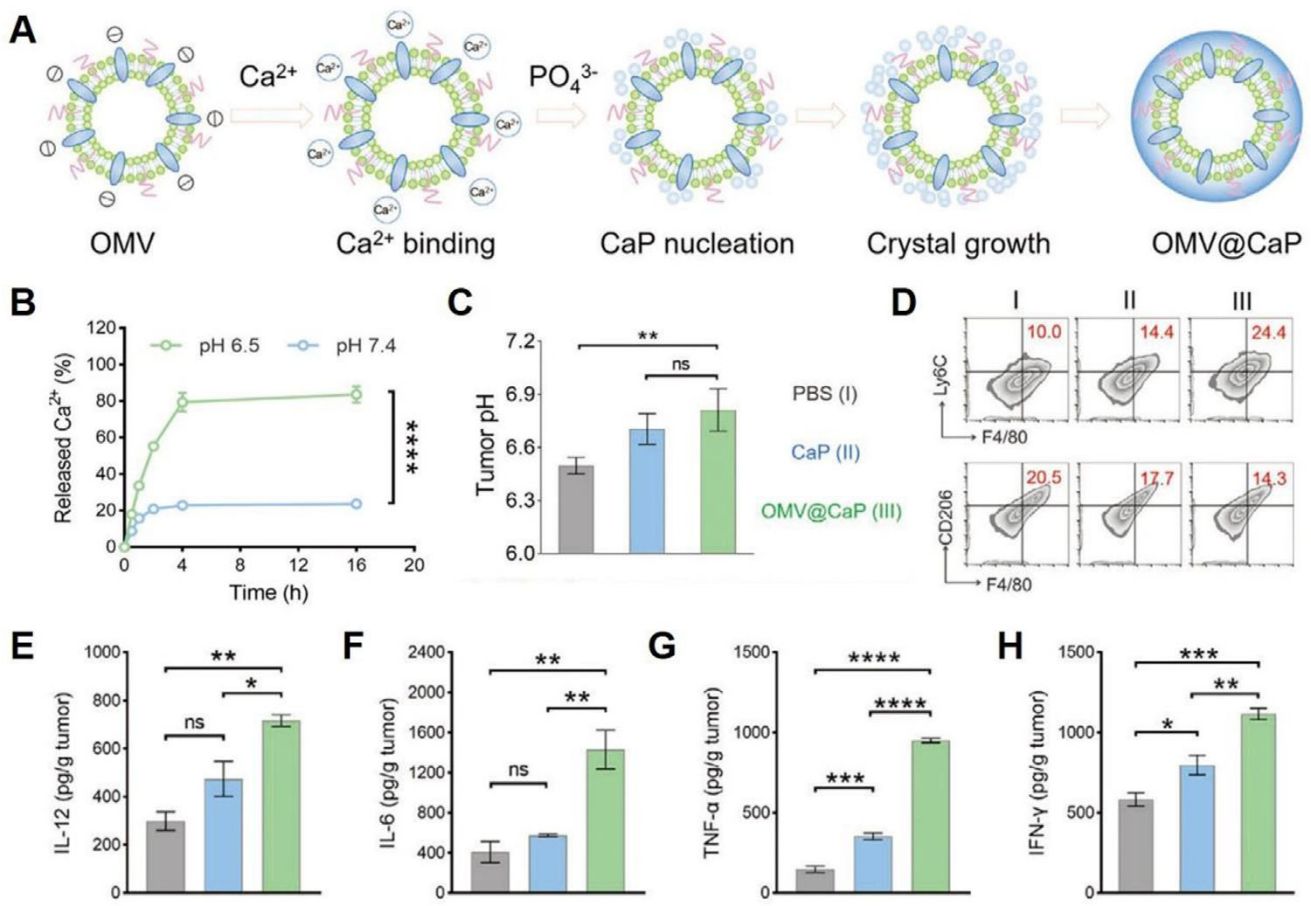
Ca^2+^ regulation of macrophage M1 polarization. (A) Schematic illustration of the synthesis of OMV@CaP. (B) Ca^2+^ release profile at different pH values. (C) pH value changes inside tumour tissues after different treatments. (D) Macrophage polarization analysis by flow cytometry. Intratumoural (E) IL‐12, (F) IL‐6, (G) TNF‐α, and (H) IFN‐γ expression were examined by ELISA. Reproduced with permission.^[^ ^123^ ^]^ Copyright 2020, John Wiley & Sons.

Tumour‐associated macrophages (TAMs) predominantly display an anti‐inflammatory M2 macrophage phenotype in the TME, which facilitates immune evasion and metastasis of tumour cells.^[^ ^117^, ^118^ ^]^ To optimize tumour immunotherapy, it is imperative to reprogram the macrophage phenotype from M2 to pro‐inflammatory M1. Various signals, including chemical and mechanical stimuli, can regulate macrophage polarization.^[^ ^119^ ^]^ Notably, Ca^2+^ levels have been implicated in the regulation of macrophage phenotype through regulating the phosphorylation of protein kinase or blocking Ca^2+^ outflow to inhibit M1 polarization of macrophages.^[^ ^120^, ^121^ ^]^ In this regard, Ca^2+^‐based bionanomaterials have been developed to manipulate intracellular Ca^2+^ levels for regulating macrophage polarization phenotypes.^[^ ^122^ ^]^ For example, Qing et al. developed a bionanomaterial consisting of bacterial outer membrane vesicles (OMV) mineralized with CaP (OMV‐CaPs) (Figure 19A),^[^ ^123^ ^]^ which effectively alleviated systemic inflammation caused by OMVs. The results revealed that the pH‐sensitive CaP shells could be dissolved to release Ca^2+^ over time at a weakly acidic pH (Figure 19B). Moreover, acidic‐pH‐induced Ca^2+^ release could neutralize the acidic pH of tumours (Figure 19C). In addition, CaP alone could induce M2‐M1 polarization, while OMV‐CaPs further improved the M1/M2 ratio (Figure 19D) and elevated intratumoural cytokine secretion including IL‐12, IL‐6, TNF‐α, and IFN‐γ) (Figure 19E–H), indicating that Ca^2+^‐based nanomaterials could participate in immune cell reprogramming, promote anti‐tumour immune response, and improve the anti‐tumour effect.

## 4 CONCLUSIONS AND FUTURE OUTLOOKS

Metal ions play a vital role in various biological processes such as cell metabolism, proliferation, migration, and death. Intracellular ion imbalances can lead to abnormal cell function and even cell death. Therefore, interfering with ion homeostasis is an attractive strategy to avoid systemic toxicity. Ion interference therapy is a promising and effective antitumour technology that has gained increasing attention due to its biosafety and lack of drug resistance. In particular, Ca^2+^ is crucial for cell survival and death, and tumour cells are more sensitive to changes in Ca^2+^ levels than normal cells. Therefore, researchers are exploring this exciting field to gain new insights into designing materials for cancer treatment.

This review delves into a range of nanoplatforms that have been developed to interfere with Ca^2+^ homeostasis for combined tumour therapy. These nanoplatforms can be classified into two categories: Ca^2+^ overload therapy and Ca^2+^ inhibition therapy. Notably, the regulation of Ca^2+^ homeostasis has the potential to target various cells, including cancer cells and immune cells, to initiate or enhance therapeutic outcomes (Table 1). These designs have been developed based on a comprehensive understanding of the deleterious impact of Ca^2+^ in tumours. The efficacy of Ca^2+^ overload has been demonstrated when used in conjunction with other therapies, and Ca^2+^ inhibition therapy has yielded remarkable results in treating drug‐resistant tumours. Moreover, given that Ca^2+^ plays a critical role in immune regulation, current research is exploring the potential of Ca^2+^ inducers as a new approach to immunotherapy. By utilizing these nanoplatforms, researchers can develop novel and effective strategies for the treatment of cancer.

**TABLE 1:** A summary of the therapeutics performance of Ca^2+^‐based biomaterials.

Host materials	Name	Therapeutics	Ref.
CaCO_3_	CaCO_3_‐IDO‐1(denoted AIM NPs)	Nanocarrier for controlled release, neutralize tumour acidity	[37]
	mPEG–PEI–AuNRs and mPEG–PEI/CaNPs	Generate carbon dioxide (CO_2_) bubbles to enhance photoacoustic (PA) signals	[41]
	CaCO_3_@COF‐BODIPY‐2l@GAG	Ca^2+^ overload, PDT	[46]
	CuS@CaCO_3_‐PEG	Ca^2+^ overload	[133]
	Cu_2_O@CaCO_3_	Ca^2+^ overload, PTT, PDT, CDT	[134]
	CaCO_3_‐ALG MSs	Ca^2+^ overload, artery‐blocking, immunoenhancement	[135]
	Alg‐CaCO_3_‐PDA‐PGED (ACDP)	Gene carriers with pH‐responsive degradation, dual‐modal US and photoacoustic (PA) imaging	[136]
	BSO‐TCPP‐Fe@CaCO_3_‐PEG	Ca^2+^ overload, oxidative stress, SDT	[137]
	CaNP_CAT+BSO_@Ce6‐PEG	Ca^2+^ overload, PDT	[138]
	^PEG^CaCUR	Ca^2+^ overload, ICD	[29]
	M@CaCO_3_@KAE NPs	Ca^2+^ overload	[130]
	CaNMs	Ca^2+^ overload, Pyroptosis (GSDME cleavage), immunotherapy	[139]
	OVA@CaCO_3_	Neutralize tumour acidity, induce autophagy of DCs, promote DAMPs release	[30]
	OVA@NP	Promote lysosome escape‐mediated antigen cross‐presentation, induce autophagy	[140]
	OVA/CaCO_3_/PLY	Enhance the immunogenicity, promote lysosome escape‐mediated antigen cross‐presentation	[141]
CaO_2_	CMFO	Ca^2+^ overload, oxidative stress	[142]
	CaO_2_‐Lbuthionine sulfoximine (BSO)‐ chloroperoxidase (CPO) (denoted BHCHC NC)	Ca^2+^ overload, oxidative stress, enzyme dynamic therapy (EDT)	[50]
	LA‐CaO_2_@PDA	Ca^2+^ overload, PTT, NO aggravate the abnormal retention of Ca^2+^, ICD	[80]
	CaO_2_‐Cu/ICG@PCM	Ca^2+^ overload, oxidative stress, CDT, PDT	[143]
	CaO_2_/Tf/CUR	Ca^2+^ overload, ferroptosis	[57]
	SH‐CaO_2_ NPs	Ca^2+^ overload, oxidative stress	[144]
	CaO_2_@TA‐FeIII	Ca^2+^ overload, oxidative stress, CDT	[53]
	PCN‐224‐CaO_2_‐HA	Ca^2+^ overload, PDT	[145]
	Fe‐GA/CaO_2_@PCM	Ca^2+^ overload, oxidative stress, PTT/CDT	[146]
	PLT@MCC/CUR	Ca^2+^ overload	[147]
	CaO_2_/Cu‐ferrocene	Ca^2+^ overload, oxidative stress	[148]
	HPB‐CaO_2_ (HC)	Ca^2+^ overload, ferroptosis	[149]
	SA‐CaO_2_	Ca^2+^ overload, PDT, tumour calcification, neutralize tumour acidity	[150]
	CaO_2_@ZIF‐Fe/Ce6@PEG(CaZFCP)	Ca^2+^ overload, oxidative stress, CDT, PDT	[55]
	CaO_2_@ZIF‐8/DOX@HA	Ca^2+^ overload, oxidative stress, chemotherapy	[151]
	CaO_2_@Mn‐PDA	Ca^2+^ overload, oxidative stress, PTT, CDT, hypoxia relief	[152]
	CaO_2_‐CuO_2_@HA NC	Ca^2+^ overload, CDT, tumour calcification	[54]
	DCC‐HA NCs	Ca^2+^ overload, tumour calcification, enzyme dynamic therapy (EDT)	[153]
	CaNP@cAD‐PEG	“Ca^2+^ interference” induced reset M2‐like TAMs to M1 phenotype, induce ICD	[154]
CaH_2_	nano‐CaH_2_	Ca^2+^ overload, neutralize tumour acidity, hydrogen therapy, immunomodulation	[60]
CaP	TiO_2_@CaP	Ca^2+^ overload, SDT	[155]
	lipid‐CaP‐Cu^2+^‐disulfiram (DSF)(Cu‐LCP/DSF NPs)	CaP as nanocarrier, ICD	[62]
	CaBPs	Ca^2+^ overload, changing the osmotic pressure	[156]
	Fe/Se‐CaP	Boost adaptive immune response	[113]
	CM@CaP NGs	Increase the delivery and uptake efficiency of antigens, induce immune responses	[27]
	OMV@CaP	Neutralize tumour acidity, M2‐to‐M1 polarization	[123]
CaS	ZnPP@PAA‐CaS	Ca^2+^ overload, tumour calcification, induce ICD	[71]
CaF_2_	CaF_2_	Ca^2+^ overload, peroxidase (POD)‐mimicking activity by generating ROS	[76]
	CaF_2_:Eu NPs	Carrier, scintillator, Ca^2+^‐induced radiosensitization	[157]

Compared to traditional therapies that use drugs to control Ca^2+^ levels, these burgeoning nanoplatform‐based strategies for regulating Ca^2+^ homeostasis have several advantages. Firstly, nanomaterials can be precisely targeted to reduce the adverse effects of Ca^2+^ on normal cells and tissues. Passive targeting, which exploits the distinctive characteristics of TME, such as the enhanced permeability and retention (EPR) effect, can facilitate the accumulation of nanomaterials at the tumour site. Additionally, nanomaterials can be actively targeted by binding to specific molecules, such as antibodies or ligands, for improved selectivity. Secondly, nanoplatforms offer stability and controllability, as their physicochemical structure can be tailored to prevent Ca^2+^ degradation before reaching the tumour site and promote nanomaterial aggregation at the target location for controlled release. This ensures sustained therapeutic efficacy of Ca^2+^ and optimized delivery to the tumour. Finally, nanomaterials can be synergistically combined with other therapeutic modalities such as PTT, PDT, SDT, and chemotherapy drugs, to enhance their antitumour efficacy. The combination of different therapies using nanoplatforms offers a synergistic effect that results in enhanced efficacy against cancer. In summary, the use of nanomaterials for regulating Ca^2+^ homeostasis represents a promising strategy for treating cancer that surpasses conventional therapies.

Despite the significant progress made in the treatment of Ca^2+^‐related tumours, there remain several challenges that need to be addressed to further enhance therapeutic outcomes. First, inadequate delivery of Ca^2+^ to target areas may result in the distribution of Ca^2+^ levels throughout the body, potentially leading to various side effects such as cardiac arrest and renal injury. The ubiquitous existence of Ca^2+^ signalling calls for precise therapeutic targets to avoid damage to normal cells. Therefore, the development of specific substrates that can precisely target tumours while avoiding uptake by normal cells and only activating within the TME is crucial. Current research focuses on surface functionalization to enhance target efficiency and routinely tests the cell viability to normal cells in vitro as well as systemic toxicity and immune responses in mice in vivo. However, these approaches are insufficient for a comprehensive assessment of biosafety and further exploration is needed in future research. Therefore, the development of specific substrates that can precisely target tumours while avoiding uptake by normal cells and only activating within the TME is crucial. Morphological regulation^[^ ^124^, ^125^, ^126^ ^]^ and surface modification^[^ ^127^, ^128^ ^]^ of nanomaterials have been found could enhance the cellular internalization capacity and better target cancer cells rather than normal cells, which is beneficial to improve biosafety and promote further clinical applications. However, detailed studies on how the morphology of Ca^2+^‐based nanomaterials modulates intracellular Ca^2+^ overload are still lacking. Moreover, extra efforts are required to systematically investigate the long‐term toxicological effects including pharmacokinetics (absorption, distribution, metabolism, and excretion), biocompatibility and toxicity, and immunogenicity in mice and even in large animal models. This is essential to achieve safe and versatile Ca^2+^‐based nanomaterials for clinical translation.

Second, It is well known that the main barrier to efficient Ca^2+^ homeostasis destroyer is the self‐regulatory pathways cells itself to buffer the intracellular calcium concentration. Once the intracellular Ca^2+^ concentration becomes overloaded, the excess Ca^2+^ can be pumped out by the plasma membrane calcium pump. Alternatively, mitochondria and the ER can serve as Ca^2+^ reservoirs and absorb Ca^2+^ to buffer cellular Ca^2+^ levels.^[^ ^42^, ^43^ ^]^ As a result, monotherapy focusing solely on Ca^2+^ overload may not achieve satisfactory therapeutic effects. Combining Ca^2+^‐based nanomaterials with other Ca^2+^ inducers could present a strategic opportunity to improve cancer treatment efficacy by regulating Ca^2+^ homeostasis changes. For instance, lonafarnib, known to activate caspase family proteins and trigger ER stress to release Ca^2+^,^[^ ^129^ ^]^ can be combined with Ca^2+^‐based nanomaterials that act as delivery vehicles to achieve a more potent disruption of Ca^2+^ homeostasis. The same approach can be applied to other Ca^2+^ efflux inhibitors, such as Ca^2+^ channel inhibitors (CUR, T‐type calcium channel blockers). Likewise, Ca^2+^ influxes like kaempferol‐3‐O‐rutinoside (KAE) and capsaicin (CAP) can be integrated with Ca^2+^‐based nanomaterials.^[^ ^130^, ^131^ ^]^ Furthermore, Ca^2+^‐based nanomaterials can be combined with other therapeutic modalities, such as PTT, PDT, SDT, US, and gas therapies, to obtain synergistic antitumour efficacy. PTT can activate the TRPV1 to promote Ca^2+^ influx.^[^ ^131^ ^]^ PDT and SDT can disrupt the Ca^2+^ pool in mitochondria, impair cell calcium buffering capacity and amplify the cell damage caused by Ca^2+^ overload.^[^ ^78^ ^]^ US, an exogenously physical stimulus, has been used to induce Ca^2+^ influx.^[^ ^29^ ^]^ NO molecules can stimulate Ca^2+^ release from the ER by activating cellular channels, leading to intracellular Ca^2+^ overload.^[^ ^80^ ^]^ H_2_S molecules also could down‐regulate the permeability of the Ca^2+^ channel and inhibit Ca^2+^ efflux.^[^ ^132^ ^]^

Third, a simplistic approach to Ca^2+^ interference therapy may not yield the desired therapeutic outcome, necessitating consideration of other metal ions’ involvement in cellular function. The combination of Ca^2+^ with other ions can potentiate ion therapy's efficacy. Last, given the pivotal role of Ca^2+^ in immune regulation, further investigation into its underlying mechanisms is imperative for achieving clinical translation and efficacy. Overall, achieving a balance between biosafety and therapeutic efficacy is crucial in utilizing nanoplatforms to modulate Ca^2+^ levels for cancer therapy. In the future, combining Ca^2+^‐based nanomaterials with other Ca^2+^ inducers could offer a strategic opportunity to improve cancer treatment efficacy through Ca^2+^ homeostasis changes. These nanomaterials have the potential to serve as delivery vehicles for specific genes, such as siRNAs, to modulate Ca^2+^ concentrations at the tumour site and impede tumour growth. Considering the remarkable biological functions of Ca^2+^, novel approaches for regulating Ca^2+^ in cancer therapy are expected to emerge. Collaborative efforts across disciplines such as biochemistry, oncology, and materials science will be crucial in devising more efficacious Ca^2+^‐based nanomaterials that can augment.

## CONFLICT OF INTEREST STATEMENT

The authors declare no conflicts of interest.
